# miR-10b*, a master inhibitor of the cell cycle, is down-regulated in human breast tumours

**DOI:** 10.1002/emmm.201201483

**Published:** 2012-11-02

**Authors:** Francesca Biagioni, Noa Bossel Ben-Moshe, Giulia Fontemaggi, Valeria Canu, Federica Mori, Barbara Antoniani, Anna Di Benedetto, Raffaela Santoro, Sabrina Germoni, Fernanda De Angelis, Anna Cambria, Roi Avraham, Giuseppe Grasso, Sabrina Strano, Paola Muti, Marcella Mottolese, Yosef Yarden, Eytan Domany, Giovanni Blandino

**Affiliations:** 1 Translational Oncogenomic Unit, Regina Elena National Cancer InstituteRome, Italy; 2 Department of Physics of Complex Systems, Weizmann Institute of ScienceRehovot, Israel; 3 Molecular Chemoprevention Group, Regina Elena National Cancer InstituteRome, Italy; 4 Department of Pathology, Regina Elena National Cancer InstituteRome, Italy; 5 SAFU Department, Regina Elena National Cancer InstituteRome, Italy; 6 Department of Oncology, Division of Pathology, S. Vincenzo HospitalTaormina, Italy; 7 Broad Institute of MIT and HarvardCambridge, MA, USA; 8 Department of Oncology, Juravinski Cancer Center McMaster University HamiltonOntario, Canada; 9 Department of Biological Regulation, Weizmann Institute of ScienceRehovot, Israel

**Keywords:** breast cancer, cell proliferation, expression profiling, microRNA, miR-10b*

## Abstract

Deregulated proliferation is a hallmark of cancer cells. Here, we show that microRNA-10b* is a master regulator of breast cancer cell proliferation and is downregulated in tumoural samples *versus* matched peritumoural counterparts. Two canonical CpG islands (5 kb) upstream from the precursor sequence are hypermethylated in the analysed breast cancer tissues. Ectopic delivery of synthetic microRNA-10b* in breast cancer cell lines or into xenograft mouse breast tumours inhibits cell proliferation and impairs tumour growth *in vivo*, respectively. We identified and validated *in vitro* and *in vivo* three novel target mRNAs of miR-10b* (BUB1, PLK1 and CCNA2), which play a remarkable role in cell cycle regulation and whose high expression in breast cancer patients is associated with reduced disease-free survival, relapse-free survival and metastasis-free survival when compared to patients with low expression. This also suggests that restoration of microRNA-10b* expression might have therapeutic promise.

## INTRODUCTION

MicroRNAs (miRs) are evolutionarily conserved, small non-coding RNA molecules that negatively regulate gene expression at the post-transcriptional level, binding through partial sequence homology to the 3′-untranslated region (3′-UTR) of target mRNAs and causing translational inhibition and/or mRNA degradation (Bartel, 2004). Several studies have indeed demonstrated that miRs are highly specific for tissue and developmental stages. Computational analyses indicate that each miR can regulate hundreds of genes, thus influencing critical cellular processes such as differentiation, cell growth, stress response and cell death (Miska, 2005; Zamore & Haley, 2005). Recent studies have demonstrated that aberrant expression of miRNA genes can be associated with different pathologies including cancers (Calin & Croce, 2006; Croce, 2009), suggesting that miRs have a potential role as oncogenes or tumour suppressor genes (Cimmino et al, 2005; Johnson et al, 2005; Voorhoeve et al, 2006).

Deciphering the molecular mechanisms involved in breast tumourigenesis has been the subject of extensive research in last years; yet unpredictable response and development of resistance to adjuvant therapies remain major challenges in the management of breast cancer patients. The power of miR expression signatures has emerged recently from several studies. Normal and breast tumour tissues can be discriminated by a miR signature as reported by Iorio et al (Iorio et al, 2005) and recent findings have also linked deregulated miR expression to breast cancer metastasis (Huang et al, 2008; Martello et al, 2010; Tavazoie et al, 2008). In addition to being potential diagnostic markers, miRs were also associated with clinical and pathological features and outcome in different tumour types (Calin et al, 2005; Iorio et al, 2008; Shell et al, 2007; Takamizawa et al, 2004; Yanaihara et al, 2006). These results suggest that altered miR expression may be important for the pathogenesis of breast cancer.

In our study, we measured miR expression profiles from 56 matched tumour and peritumoural breast cancer tissues as well as from 5 tumour and 3 peritumour unmatched samples. Our aim was to identify new miRs involved in the process of neoplastic transformation.

The microarray data analysis identified, in every subgroup of breast cancers analysed, a specific subset of miRs that were differentially expressed in tumour compared to peritumoural tissues. We found that microRNA-10b* was downregulated in tumoural samples of all three types when compared to their matched peritumoural counterparts. This is due to hypermethylation of CpG islands found upstream from the miR10b/10b* locus.

A miR can be derived from each arm of the pre-miR hairpin. The less common of these two miR products is identified by the * next to the miR name (Bhayani et al, 2012). The more widely characterized miR derived from this precursor is miR-10b. Overexpression of miR-10b in non-metastatic breast tumours leads to tumour invasion and distant metastasis in xenotransplantation models (Ma et al, 2007). Conversely, silencing of miR-10b expression suppresses metastasis in a mouse mammary tumour model (Ma et al, 2010). On the other hand, very little is known about the role of miR-10b* in breast transformation. We found that miR-10b* controls cell cycle progression and proliferation by targeting the expression of BUB1, PLK1 and CCNA2, and that low expression levels of these three genes are, to various extents, predictive of less aggressive disease and of longer relapse- and metastasis-free survival. Finally, we showed that intratumoural delivery of miR-10b* impaired breast xenograft tumour growth. This is accompanied by reduced expression of BUB1, PLK1 and CCNA2 proteins and proliferation markers.

## RESULTS

### Deregulation of miR expression between tumoural and peritumoural breast cancer tissues

In exploring the potential involvement of miRs in breast tumourigenesis, we profiled the miR expression of 64 primary breast cancer patients, comprising 56 matched tumour and adjacent peritumoural breast tissues as well as 5 tumour and 3 peritumour unmatched tissues, using the Agilent microarray platform. The pathological characteristics of the samples collection are summarized in Supporting Information Table S1.

**Figure 1:**
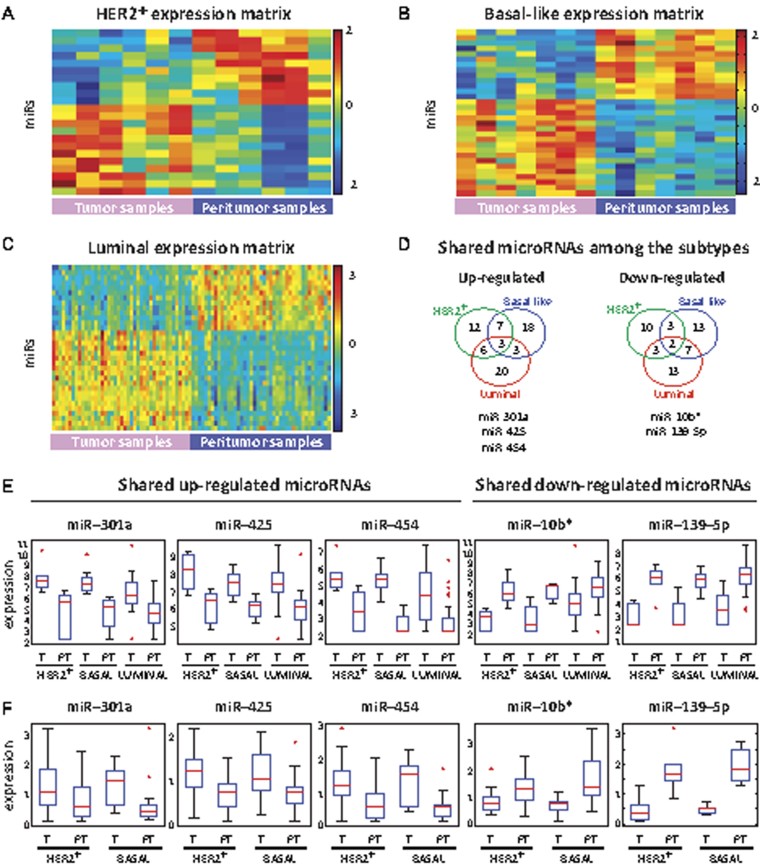
Expression profiles of the miRs, which differentiate breast tumour from matched peritumour samples **A–C.** SPIN-ordered (see Materials and Methods section) expression matrix of the miRs differentially expressed between HER2^+^ (panel a, 22 miRs), Basal-like (panel b, 31 miRs) and Luminal (panel c, 33 miRs) breast tumours and their matched peritumour samples. Colours indicate expression levels after centering and normalizing each miR (row), with red denoting relatively high expression and blue relatively low expression.**D.** Venn diagram showing the numbers of shared and not shared differentiating miRs between the various subtypes of breast cancer. miRs that are up (down) regulated in the tumour tissue are shown on the left (right).**E.** Box-plots of the expression levels (log base 2 scale) of the five shared miRs in tumour (T) and peritumour (PT) samples in each breast cancer subtype.**F.** RT-qPCR analysis of the five-shared miRs in matched tumour (T) and peritumour (PT) samples of HER2^+^ and Basal-like subtypes in a second cohort of 32 representative patients (16 HER2^+^ and 16 Basal-like patients).

Several statistical analyses were combined (including noise estimation, *t*-test and comparison of matched sample pairs, see Materials and Methods section) in order to identify a subset of miRs significantly differentially expressed between tumour and peritumour samples, done separately for each of the three breast cancer subgroups studied (Fig 1A–C). We identified 22 differentially expressed miRs in HER2-overexpressing (HER2^+^) tumours, 31 in Basal-like tumours and 33 in Luminal tumours (Supporting Information Tables S2 and S3A–S3C). Among these miRs, we identified two (miR-10b* and miR-139-5p) that were down-regulated and three (miR-425, miR-454 and miR-301a) that were up-regulated for all three subtypes (Fig 1D and E and Supporting Information Fig S1A). To evaluate the reliability of our results, we first validated by RT-qPCR the up- and down-regulation of these five-shared miRs in a small group of samples derived from the same patient cohort (Supporting Information Fig S1B). Next, we validated our results by RT-qPCR in an independent set of HER2^+^ and Basal-like tumour samples and their matched peritumoural tissues (Fig 1F and Supporting Information Table S4). Thus, up-regulation of miR-425, miR-454 and miR-301a and down-regulation of miR-10b* and miR-139-5p were definitively confirmed.

These results indicate that specific modifications in the miR expression pattern are characteristic of human breast cancer tissue when compared to their matched peritumoural samples. Furthermore, there is a group of miRs that is similarly deregulated in all three major subgroups of breast cancer.

### CpG island hypermethylation contributes to microRNA-10b* down-regulation in breast cancer samples

Downregulation of miRs has generally been observed in various types of human cancer, including breast cancer. This might suggest that some of these miRs act as tumour suppressor genes (TSGs; Kumar et al, 2007; Lu et al, 2005; Ozen et al, 2008). Since the down-regulation of many TSGs in human cancer has been frequently linked to the hypermethylation of CpG sites located within their promoter regions, the same mechanism may play an important role in silencing tumour suppressor miRs in tumours.

**Figure 2:**
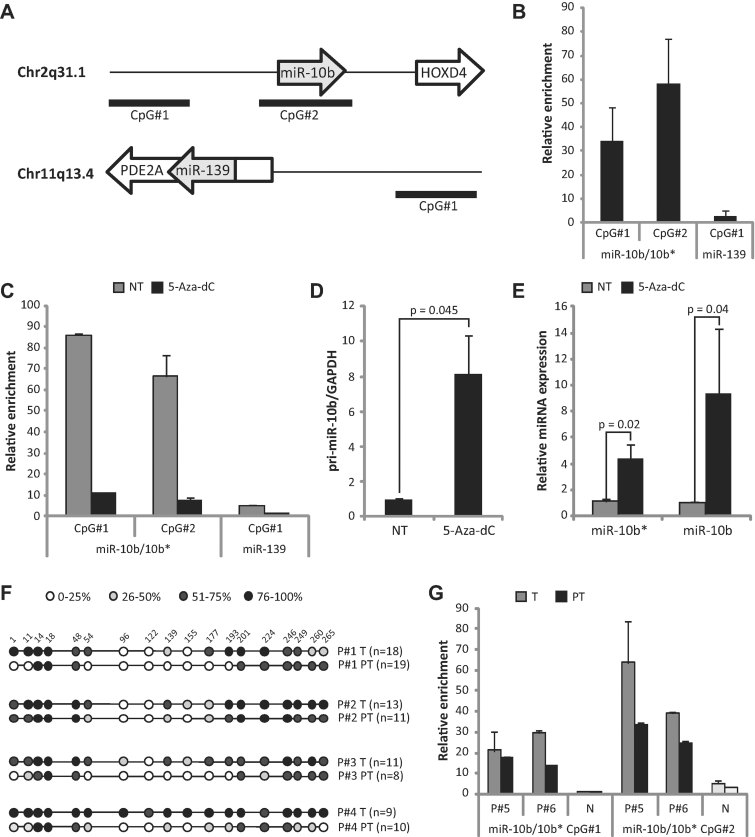
miR-10b locus is hypermethylated in breast cancer **A.** Schematic representation of the miR-10b and miR-139-5p genomic loci. The analysed CpG islands are indicated (solid lines).**B.** MeDIP analysis on breast cancer cell lines was performed as described in the text. Immunoprecipitated genomic DNA from MCF7 cell line was quantified by using qPCR with TaqMan assays directed to miR-10b* CpG islands #1 and #2 and miR-139-5p CpG Island #1.**C.** MeDIP experiment of MCF7 cells treated with 5-aza-dC. The percentage of enrichment over the INPUT of both CpG island #1 and #2 diminishes in the cells treated with the demethylating agent. NT = not treated cells.**D,E.** RT-qPCR analysis of pri-miR-10b (**D**), mature miR-10b* and miR-10b (**E**) expression was performed in MCF7 breast cancer cells treated with the demethylating agent 5-aza-dC *versus* untreated cells (NT).**F.** Bisulphite sequencing results from four matched tumoural and peritumoural breast patient samples. Results were averaged for each CpG position, whereby the number of investigated clones is presented between parenthesis and the shading of each circle represents the percentage of methylation as indicated. Relative positions of nucleotides inside the CpG islands are indicated.**G** MeDIP experiments performed on genomic DNA extracted from breast cancer tissues. MeDIP were carried out with antibody directed against 5-methylcitidine in two breast cancer and two reductive mammoplasty samples. N = reductive mammoplasty. The immunoprecipitated genomic DNA was quantified by qPCR using TaqMan assays amplifying miR-10b* CpG Island #1, CpG Island #2 and miR-139-5p CpG Island #1. Relative enrichments are presented as percentages of the input DNA used for the immunoprecipitations.

To investigate whether the down-regulation of miR-10b* and miR-139-5p could be attributable to hypermethylation events occurring during breast carcinogenesis, we first examined the genomic loci of miR-10b* and miR-139-5p for the presence of CpG islands (Supporting Information Fig S2A and S2B). Given that miR-10b* is an intergenic miR, we analysed a 5 Kb-long region upstream from the miR-10b* precursor coding sequence using the UCSC Genome Browser. We found two CpG islands located 2659 bp and 82 bp upstream the precursor sequence, respectively (Fig 2A). miR-139-5p is an intragenic miR located in the *PDE2A* gene and consequently the 5 kb upstream *PDE2A* transcription start site and the entire gene sequence were evaluated. We analysed one intragenic CpG island located at 27,116 bp upstream from the miR genomic precursor sequence. To evaluate whether these CpG islands undergo methylation, the genomic DNA extracted from MCF7 cells was immunoprecipitated with an antibody directed to 5-methylcytosine (5mC). As shown in Fig 2B, both CpG islands upstream of miR-10b* were found to be methylated. Very low levels of methylation were observed on the CpG island upstream of miR-139-5p (Fig 2B). This could suggest that other epigenetic mechanisms might underlie its down-regulation in breast tumours. Of note, methylation of miR10b/10b* CpG islands was abrogated when 5-Aza-2′-deoxycytidine (5-aza-dC), a demethylating agent, was added to the MCF7 cell culture (Fig 2C). There was almost no effect of the treatment on the analysed miR-139 CpG island (Fig 2C). The expression analyses demonstrated that the microRNA-10b* precursor (Fig 2D), as well as the mature miR-10b and -10b* (Fig 2E), are induced in MCF7 cells following 5-aza-dC pharmacological treatment. This finding is in line with the methylation status of CpG islands upstream of miR-10b*. 5-aza-dC treatment did not affect the expression of the three up-regulated miRs (miR-425, miR-454 and miR-301a; Supporting Information Fig S2C). To provide further evidence to the role of CpG island methylation to the down-regulation of miR-10b* expression, we investigated the methylation status of the two CpG islands mentioned above using genomic DNA derived from six matched tumour and peritumoural breast tissues. Bisulphite conversion followed by sequencing revealed that CpG island #2 displays a significant (*p* = 0.035) higher overall degree of methylation (69.6%) in four tumour specimens, when compared to their matched peritumour samples (31.6%; Fig 2F). As shown in Fig 2G, MeDIP analysis demonstrated a higher methylation degree of both CpG islands located upstream to miR-10b* in the tumour samples in comparison to matched peritumour samples. MeDIP analysis performed on genomic DNA extracted from unmatched mammary healthy tissues of two reductive mammoplasties exhibited very low levels of methylation for both CpG#1 and GpG#2 islands (Fig 2G). Altogether, these results indicate that methylation of CpG islands may underlie down-regulation of miR-10b* expression in breast cancer samples.

### miR-10b* inhibits the proliferation of breast cancer cell lines

**Figure 3:**
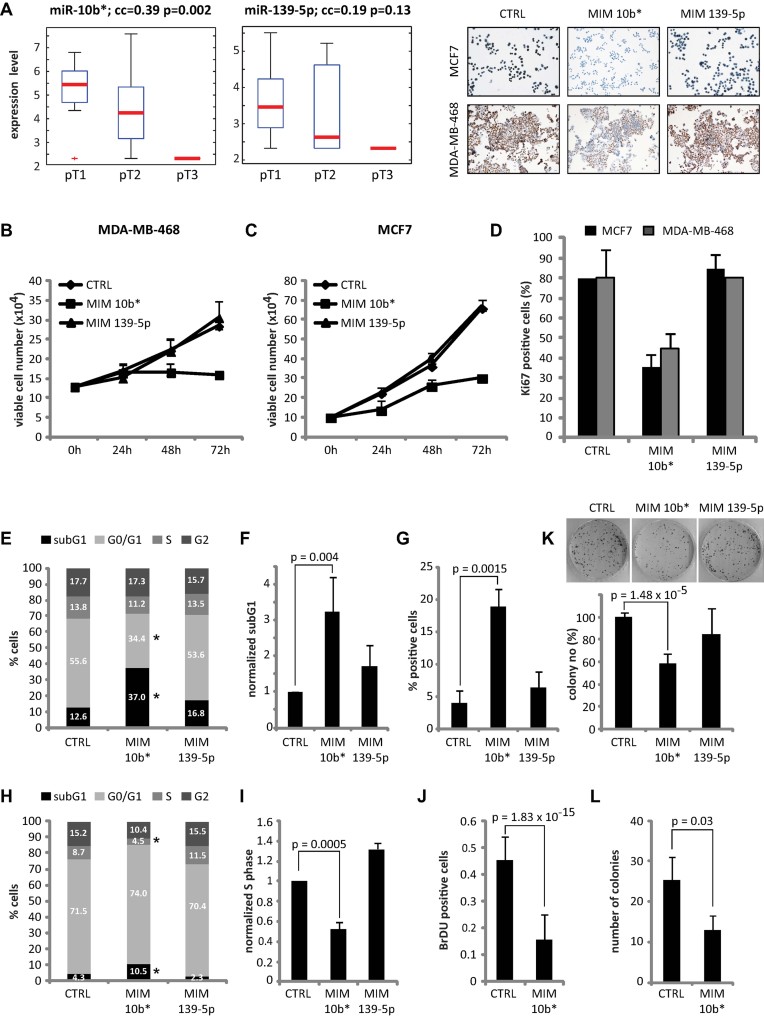
miR-10b* expression affects breast cancer cells proliferation All the histograms of the figure represent the mean of three independent experiments ± SE. The statistical significance was determined by Student's *t* test. **A.** Box-plots of the expression levels of miR-10b* and miR-139-5p for tumours of different sizes (pT1 < pT2 < pT3). The correlation of expression with tumour size was calculated by Spearman correlations (correlation-coefficient values (cc) and *p*-values (p) are indicated in each panel).**B,C.** Growth curves of MDA-MB-468 (**B**) and MCF7 (**C**) cells transfected with miR-10b* (MIM 10b*) or miR-139-5p (MIM 139-5p) or control mimic (CTRL) were performed harvesting the cells after 24, 48 and 72 h from transfection.**D.** Ki67 proliferation marker was evaluated by immunocytochemistry in MDA-MB-468 and MCF7 cells transfected with miR-10b* (MIM 10b*) or miR-139-5p (MIM 139-5p) or control mimic (CTRL). The upper panel shows representative pictures while the graph shows the percentages of Ki67-positive cells.**E–G.** Cell cycle analysis of MDA-MB-468 cells transfected with MIM 10b* or MIM 139-5p or control mimic. (**E**) The percentage of cells in subG1, G0/G1, S and G2-phase are expressed as a percentage of the total cell number. *p < 0.05. (**F**) The ratio of subG1 values of miR-10b* and miR-139-5p-transfected cells over the control mimic is shown. (**G**) The percentage of positive cells in TUNEL assay of MDA-MB-468 cells transfected with the indicated mimic molecules is shown.**H–J.** Cell cycle analysis on MCF7 cells transfected with the indicated mimic molecules. (**H**) The percentage of cells in subG1, G0/G1, S and G2-phase are expressed as a percentage of the total cell number. *p < 0.05. (**I**) The ratio of *S* values of miR-10b* and miR-139-5p-transfected cells over the control mimic is shown. (**J**) MCF7 cells overexpressing MIM 10b* or control mimic were treated with BrdU to analyse DNA synthesis. Quantification of (*BrdU*)-positive cells is shown.**K.** Colony formation assay was performed in MCF-7 cells transfected with the indicated mimic molecules by seeding 1 × 10^3^ cells in 60 mm dishes and fixing the cells after 21 days with crystal violet.**L.** Soft agar assay was performed in MCF7 cells transfected with mimic ctrl or mimic 10b* by seeding 1 × 10^4^ cells for 2 weeks.

To elucidate the biological significance of the down-regulation of miR-10b* and miR-139-5p in breast cancer, we analysed potential correlations between their expression and commonly known clinical attributes. As shown in Fig 3A, a statistically significant negative correlation was found between miR-10b* expression and tumour size (*T*). Lower levels of miR-10b* correspond to higher tumour sizes (Fig 3A). Since altered cell proliferation correlates with tumour size, we investigated the effects of miR-10b* expression on cell proliferation.

Transduction of miR-10b* mimic reduced the proliferation capacity of MCF7 and MDA-MB-468 cells when compared to the cells transduced with control mimic (Fig 3B and C). No significant variation was found in cells over-expressing miR-139-5p (Fig 3B and C). Accordingly, the percentage of Ki67-positive cells in the miR-10b*-over-expressing MCF7 and MDA-MB-468 populations was substantially lower than in control cells (Fig 3D). No differences were observed in the miR-139-5p-over-expressing population (Fig 3D).

We next analysed the effect of miR-10b* or miR-139-5p expression on cell-cycle distribution by flow cytometry. In MDA-MB-468 cells over-expressing miR-10b*, there was a significant decrease in G1 phase accompanied by an increase in the subG1 phase of the cell cycle compared to control cells (Fig 3E and F), indicating perturbation of cell cycle. Indeed, TUNEL assay performed under the same experimental conditions clearly confirmed an increase in the percentage of apoptotic cells in the miR-10b*-over-expressing population compared to control cells (Fig 3G and Supporting Information Fig S3A and S3B). In miR-10b*-over-expressing MCF7 cells, we observed a decrease in S phase accompanied by an increased sub-G1 fraction of the cell cycle compared to control cells (Fig 3H and I and Supporting Information Fig S3C). The 5-bromo-2′-deoxyuridine (BrdU) incorporation assay confirmed that miR-10b*-over-expressing MCF7 cells are less proliferating than control cells (Fig 3J and Supporting Information Fig S3D). No significant effects of miR-139-5p expression on the cell cycle were observed either in MDA-MB-468 or in MCF7 cells (Fig 3E and H).

The ability of miR-10b* expression to inhibit cell proliferation was further confirmed by colony formation assays in MCF7 cells (Fig 3K). Soft agar colony formation assays indicated that miR-10b* over-expression reduced the ability of cells to grow in an anchorage-independent manner (Fig 3L). Altogether, these results show that miR-10b* inhibits the proliferation of breast cancer cell lines.

### miR-10b* represses cell cycle regulatory genes with prognostic power in breast cancer

**Figure 4:**
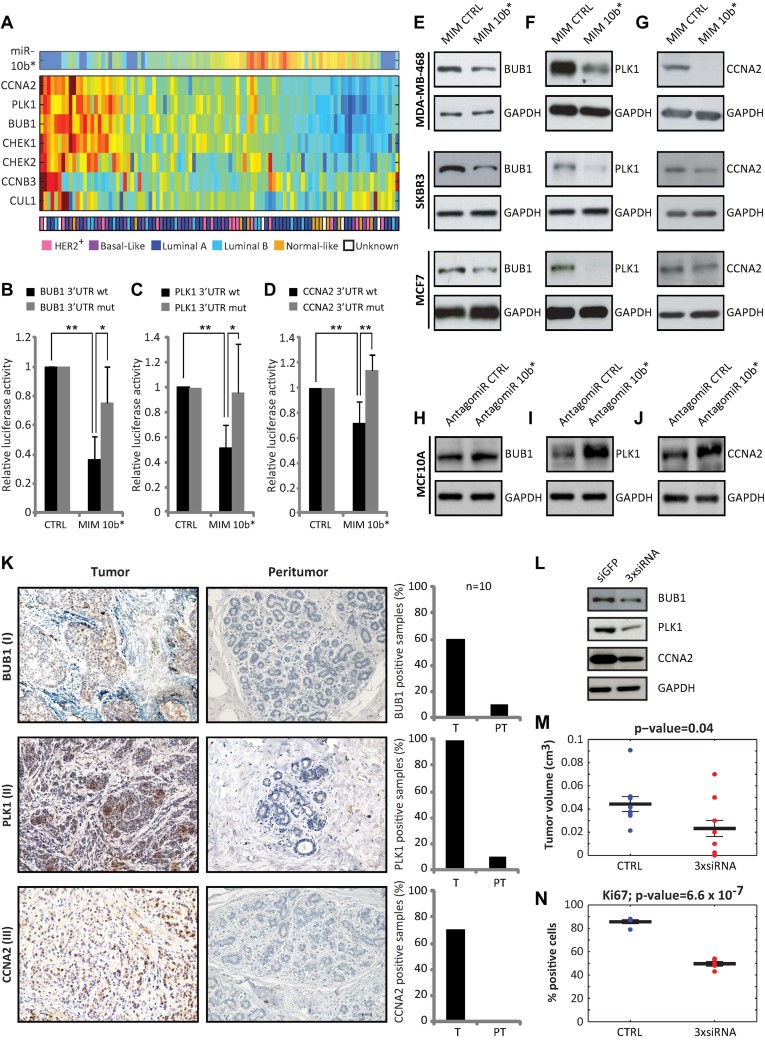
miR-10b* down-regulates the expression of BUB1, PLK1 and CCNA2 proteins in breast cancer cell lines **A.** SPIN-ordered expression matrix of miR-10b* and its seven predicted target genes connected to cell cycle, across 100 breast cancer tumours. Colours indicate expression levels after centering and normalizing each gene (row), with red denoting relatively high expression and blue relatively low expression. The colour bar at the bottom represents the breast tumour subtypes – pink for HER2^+^, purple for Basal-like, blue for Luminal A, Cyan for Luminal B, orange for Normal-like and white for unknown subtype).**B–D.** Expression vectors carrying a luciferase reporter followed by the 3′-UTR regions of BUB1 (**B**), PLK1 (**C**) or CCNA2 (**D**), in their wild-type form (black bars) or mutated in the miR-10b* complementary sequence (grey bars), were transfected in H1299 cells in the presence of mimic-10b* or control mimic. Normalized luciferase activity values from three independent experiments in triplicate are shown. *p < 0.01; **p < 0.05.**E–G.** Immunoblotting of BUB1 (**E**), PLK1 (**F**) and CCNA2 (**G**) in MDA-MB-468, SKBR3 and MCF7 cells transfected with either mimic-10b* or control mimic.**H–J.** Immunoblotting of BUB1 (**H**), PLK1 (**I**) and CCNA2 (**J**) in MCF10a cells transfected with either antagomiR-10b* or antagomiR control.**K.** Immunohistochemical analysis of BUB1, PLK1 and CCNA2 protein expression was analysed in ten representative breast cancer tissues (matched tumour and peritumour). Representative images of invasive ductal breast carcinomas (tumour) where BUB1 (I), PLK1 (II) and CCNA2 (III) show nuclear immunostaining in more than 50% of tumour cells and their relative peritumour tissues are shown (scale bar 30 µm). Right graphs depict the percentage of tissues showing immunostaining for BUB1 (upper graph) or PLK1 (middle graph) or CCNA2 (lower graph) in the group of tumours or peritumours.**L.** Immunoblotting of BUB1, PLK1 and CCNA2 in MDA-MB-468 tumours derived from triple siRNA (3xsiRNA) SCID mice.**M.** Tumour volumes measured 3 weeks after the injection. The central bold lines denote mean values, the vertical lines ± SEM (*n* = 9 mice in siGFP group and *n* = 10 in 3xsiRNA group) and the circles data points. *p*-value was calculated by two-samples *t*-test and indicated in the figure.**N.** Immunohistochemistry quantification of Ki67 (V) expression from five representative siGFP control and 3xsiRNA (siBUB1, siPLK1 and siCCNA2) mice are shown. *p*-values were calculated by two sample *t*-test, and indicated in the figure.

To unravel the molecular mechanisms through which miR-10b* exerts its tumour suppressor functions and is involved in the regulation of cell cycle and proliferation, we first searched for putative miR-10b* target mRNAs. Of the available sequence-based miR target prediction algorithms, Microcosm Targets (http://www.ebi.ac.uk/enright-srv/microcosm/htdocs/targets/v5/) is the only one that provides targets of miR-10b*. We identified 736 putative target genes and since many of these are likely to be false positives, we used a publicly available dataset (Enerly et al, 2011) that contains expression data for both miR-10b* and mRNA from 100 breast cancer patients to provide a filter for putative targets. Specifically, we calculated the correlation between the expression levels of these 736 putative target genes and those of miR-10b*. Forty-two of the predicted target genes of miR-10b* were significantly (10% FDR) anti-correlated with the miR's expression levels, and we selected these as our candidate targets. Using the DAVID tool (Dennis et al, 2003; Huang da et al, 2009), we searched for pathways enriched for these candidate targets. The cell cycle pathway was significantly enriched, much more than any other pathway, encompassing seven out of the predicted 42 genes (corresponding to <0.001% FDR; Supporting Information Table S5A and S5B). We investigated the expression levels of the seven putative target genes contained in the cell cycle pathway: the three targets among these seven genes with the most negatively correlated expression to the miR were *CCNA2*, *PLK1*, and *BUB1* (*p* < 0.0005, see Fig 4A and Supporting Information Fig S4A). These three genes were selected and subjected to detailed experimental investigations.

BUB1 is a serine/threonine protein kinase bound to kinetochores and plays a key role in the establishment of mitotic spindle checkpoint and chromosome congression (Elowe, 2011). PLK1 controls G2/M transition supporting the centrosomes' maturation in late G2/early prophase and bipolar spindle formation (de Carcer et al, 2011). CCNA2 belongs to the highly conserved cyclin family, whose members are characterized by protein abundance oscillation, and controls cell cycle in G1/S and G2/M transitions (Malumbres & Barbacid, 2009). The *BUB1*-, *PLK1*- and *CCNA2*-encode mRNA contains 3′-UTR elements that are partially complementary to miR-10b* (Supporting Information Fig S4B). To formally demonstrate that miR-10b* targets *BUB1*, *PLK1* and *CCNA2* mRNAs, the 3′-UTR of each of the three targets was cloned down-stream to the Renilla luciferase gene into the psiCHEK2 vector (Supporting Information Fig S4C). As shown in Fig 4B, miR10b* co-transfection significantly decreased the Renilla luciferase activity of the vector encoding the *BUB1* 3′-UTR. This was also observed using the *PLK1* 3′-UTR (Fig 4C) and the *CCNA2* 3′-UTR (Fig 4D). A partial recovery of the luciferase activity was observed when vectors carrying targets 3′-UTR of BUB1, PLK1 and CCNA2, with mutations in the miR-10b* complementary sequences, were tested (Fig 4B–D).

To examine the effect of miR-10b* expression on the protein levels of these targets, MCF7, MDA-MB-468 and SKBR3 cells (which have a lower level of miR-10b* expression compared with MCF10A cells, see Supporting Information Fig S4D) were transfected with miR-10b* mimic or control mimic and the protein levels of BUB1, PLK1 and CCNA2 were analysed by Western blot. As shown in Fig 4E–G, miR-10b* exogenous expression strongly reduced the amount of BUB1, PLK1 and CCNA2 protein in all cell lines. Conversely, the transfection of an inhibitor of miR-10b* activity in untransformed MCF10A cells leads to an increase of BUB1, PLK1 and CCNA2 protein levels (Fig 4H–J and Supporting Information Fig S4E and S4F).

Analysis of these miR-10b* targets by immunohistochemistry (IHC) in ten matched breast tumour and peritumoural tissues (from the cohort analysed by microarray miR profiling) showed remarkable increased staining of BUB1, PLK1 and CCNA2 in the tumour tissues (Fig 4K).

To further investigate the involvement of miR-10b* in control of proliferation through the modulation of these cell cycle regulatory proteins, we tested three different siRNA molecules for BUB1, PLK1 or CCNA2 in MCF7 cells. The knock-down of each miR-10b* target in each experimental condition tested caused a significant reduction (albeit to different extents) in the colony-forming ability of MCF7 cells (Supporting Information Fig S4G–S4L). We also subcutaneously inoculated immunodeficient SCID mice with MDA-MB-468 cells, whose expression of BUB1, PLK1 or CCNA2 proteins was concomitantly knocked-down. Before the inoculation, the transduced cells were tested for the efficiency of the silencing of BUB1, PLK1 and CCNA2 as shown in Fig 4L. Inoculated mice were sacrificed 3 weeks after cell inoculation and, as shown in Fig 4M and N, cells whose expression of BUB1, PLK1 and CCNA2 was concomitantly silenced engrafted less efficiently than those transfected with control siRNA.

**Figure 5:**
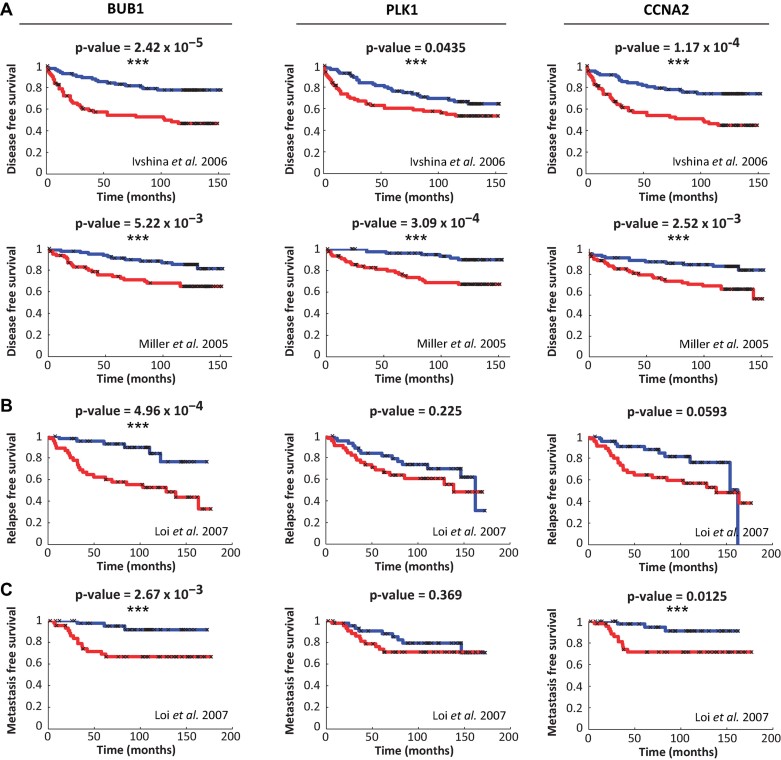
Clinical association of miR-10b* target gene expression with survival of breast cancer patients **A–C.** The association between expression levels of miR-10b* target genes (BUB1, PLK1 and CCNA2) and disease (**A**)/relapse (**B**)/distant metastasis (**C**) – free survival was evaluated by Kaplan–Meier analysis in three public datasets, Ivshina et al. (Ivshina et al, 2006), 249 patients, Miller et al. (Miller et al, 2005), 237 patients and Loi et al. (Loi et al, 2007), 135 patients. The two compared groups are the third of patients with the highest expression levels of each target gene (red) *versus* the third of patients with the lowest expression (blue). Statistically significant results (*p*-value < 0.05) are indicated by the presence of ***.

When the prognostic value of BUB1, PLK1 and CCNA2 expression was evaluated using publicly available breast cancer microarray datasets (with associated clinical information), we found that high expression levels of all three genes are predictive of lower disease-free survival in the datasets from Ivshina et al. (Ivshina et al, 2006) and Miller et al. (Miller et al, 2005; Fig 5A). A statistically significant prognostic power of BUB1 was found also in the dataset from Loi et al. (Loi et al, 2007) for both relapse-free (Fig 5B) and metastasis-free survival (Fig 5C), while in the same dataset the CCNA2 expression was associated only with onset of metastasis. A trend of association between low miR-10b* expression level and worse prognosis was also evidenced by Kaplan–Meier analysis of the Enerly et al dataset (Enerly et al, 2011; Supporting Information Fig S5).

### Intra-tumoural delivery of miR-10b* reduces tumour size in a breast cancer xenograft model

**Figure 6:**
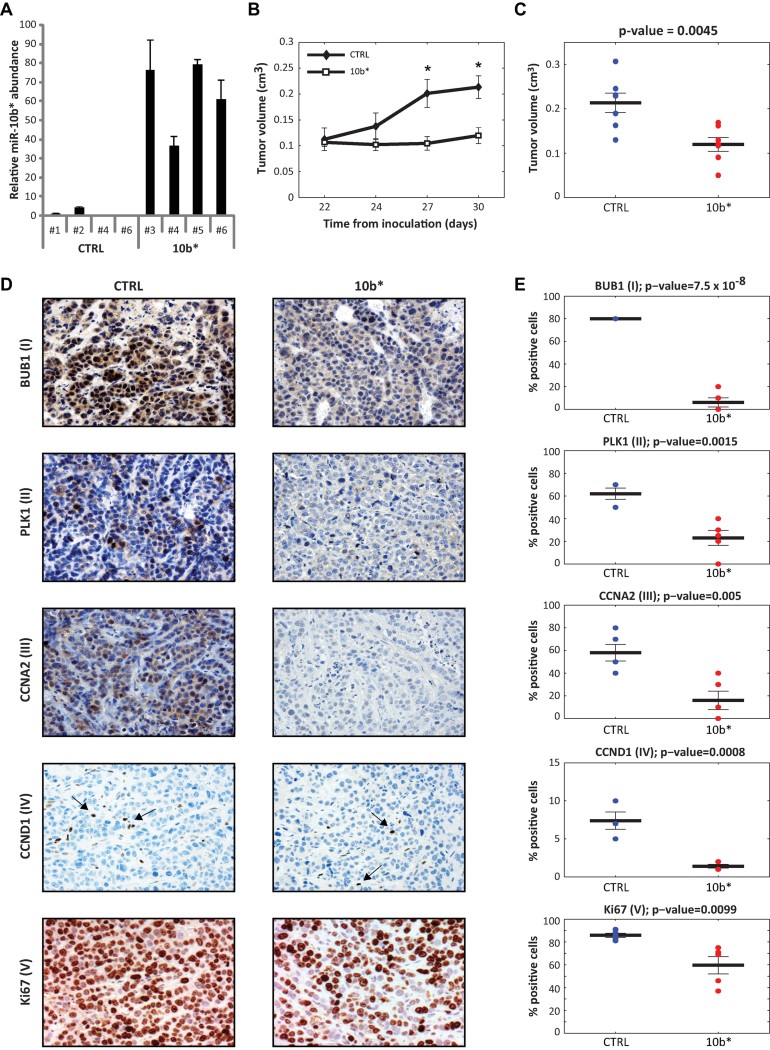
Intra-tumoural delivery of miR-10b* reduces tumour size in a breast cancer xenograft model **A.** Relative miR-10b* abundance in MDA-MB-468 tumours. Total RNA was prepared from tumours harvested 5 day post the final treatment and RT–qPCR was performed using probe specific for miR-10b*. *C*_t_ values were used to calculate absolute miR-10b* copy numbers and expressed relative to the average expression in control tumours (1.0). Four representative mice for control group (numbered on *x*-axis #1, #2, #4 and #6) and miR-10b* group (numbered on *x*-axis #3, #4, #5 and #6) were shown.**B.** A total of 10 × 10^6^ MDA-MB-468 breast cancer cells in 30% matrigel were subcutaneously injected into immunodeficient SCID mice. On days 22, 24, 27 and 30 from cells inoculation, synthetic miR-10b* or control double-stranded and ready-to-use miRs conjugated with the siPORT transfection reagent were intratumourally delivered into groups of seven animals. Caliper measurements were taken to determine the length and width of each tumour and to calculate total tumour volumes. Data is presented as mean ± SEM (*n* = 7 mice in each group). *p*-values were calculated by two-samples *t*-test; significant results are marked by * (*p*-value for day 27 = 0.0075 and *p*-value for day 30 = 0.0045).**C.** Tumour volumes measured at day 30 after the first injection. The central bold lines denote mean values, the vertical lines ± SEM (*n* = 7 mice in each group) and the circles data points. *p*-values was calculated by 2-samples *t*-test and indicated in the figure.**D,E.** Immunohistochemical analysis of BUB1, PLK1, CCNA2, CCND1 and ki67 protein expression was analysed in five representative mimic control and mimic 10b* treated mice. (**D**) Representative images and (**E**) relative quantification of BUB1 (I), PLK1 (II), CCNA2 (III), CCND1 (IV) and ki67 (V) positive cells of control and miR-10b* overexpressing tumours are shown.

To explore the therapeutic potential of miR-10b* in established tumours, we subcutaneously inoculated immunodeficient SCID mice with human MDA-MB-468 breast cancer cells. Three weeks after injection, when tumour cells had formed solid and palpable tumours with an average volume of 100 mm^3^, animals were subdivided into two groups and either treated with miR-10b* mimic or a negative control miR. The inoculations were performed administering miR-10b* mimic by four intra-tumoural injections each every 3 days. The synthetic miRs were complexed with siPORTamine (siPORT), a lipid-based transfection reagent that enhances cellular uptake of the oligonucleotide (Trang et al, 2010). All mice were sacrificed 5 days after the last injection. miR-10b* abundance was investigated in a subset of representative tumours, both miR-10b*-treated and control (Fig 6A). As shown in Fig 6B, local delivery of synthetic miR-10b* induced a specific inhibitory response and robustly interfered with tumour growth. The distribution of tumour volumes among the groups of miR-10b*-treated and control mice at the time of the last injection is shown in Fig 6C. IHC analysis for BUB1, PLK1 and CCNA2 proteins revealed a significant decrease of protein level in mice injected with synthetic miR-10b* compared to control mice [Fig 6D and E (I–III)]. Ki-67 and Cyclin D1 protein expression was significantly (Ki67 *p* = 0.009; CCND1 *p* = 0.0008) decreased in their levels in the tumours injected with miR-10b*, compared to control tumours [Fig 6D and E (IV–V)]. miR-10b*-treated tumours also showed a significantly higher number of TUNEL-positive cells than those injected with control mimic (Supporting Information Fig S6).

Overall, *in vivo* observations on the effect of miR-10b* expression in breast cancer tumours together with those observed in cell lines confirm the key role of miR-10b* in the control of breast cancer cell proliferation.

## DISCUSSION

**Figure 7:**
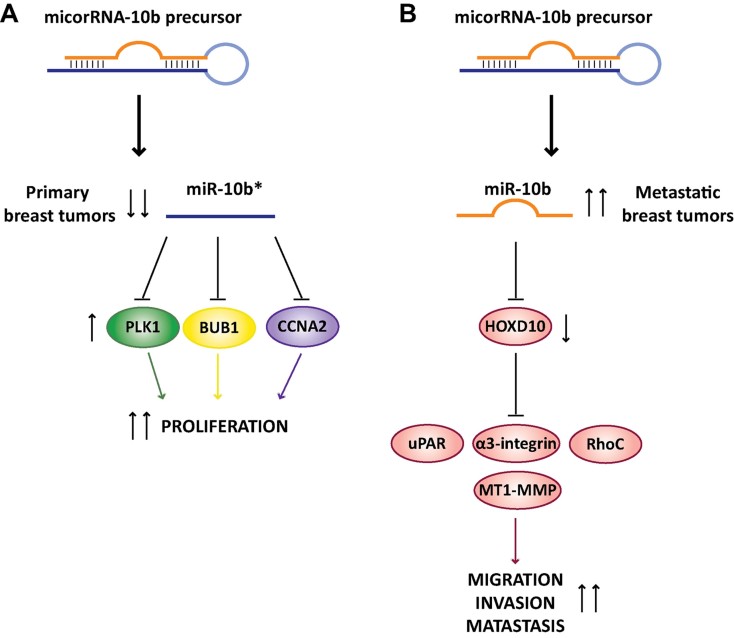
Schematic representation of working model miR-10b and miR-10b* are generated from a common precursor. In primary breast tumours, miR-10b* is down-regulated by promoter hypermethylation. This leads to upregulation of its targets genes, BUB1, PLK1 and CCNA2 that are key components of the cell cycle machinery. Such a disregulation results in aberrant tumour cell proliferation. In metastatic breast tumours, it has been reported (Ma et al, 2007, 2010) that the over-expression of miR-10b is strictly related to the invasive program that leads to metastasis formation.

In the present study, we identified miR-10b* as a master regulator of breast cancer cell proliferation. We showed that down-regulation of miR-10b* expression occurs specifically in breast tumour specimens when compared to their matched peritumoural samples. The analysed sample collection included breast cancer specimens representative of Luminal, Triple-Negative and HER2-amplified disease subtypes. miR-10b* falls among those miRs whose deregulated expression features in all of the three breast cancer subtypes. This suggests that down-regulation of miR-10b* expression might precede those oncogenic alterations, which contribute to breast cancer subtype specificity. Our findings, together with those published by Weinberg's group (Ma et al, 2007, 2010), highlight the key roles of the miR-10b locus in breast cancer establishment (miR-10b*) and spreading (miR-10b). Indeed, miR-10b is highly expressed in metastatic breast cancer as well as in other advanced tumours such as pancreatic ductal carcinoma (Bloomston et al, 2007; Preis et al, 2011) and glioblastoma tumours (Ciafre et al, 2005), while we show here that miR-10b* is down-regulated through hypermethylation of its regulatory regions in primary breast cancers. This might suggest that down-regulation of miR-10b* promotes aberrant breast cancer cell proliferation, which is later paired with increased migration, invasion and metastasis as a consequence of the transcriptional activation of miR-10b (Fig 7).

The regulation of the expression of a majority of miRs has yet to be explored. This gap in our knowledge is mainly due to the uncertainty regarding location of regulatory regions where epigenetic modifications occur and binding sites for specific transcription factors reside. Interestingly, the miR-10b/10b* locus appears to undergo both epigenetic modifications and transcriptional activation in breast tumours. Methylation of miR-10b/10b* promoter CpG islands has been recently shown in gastric cancer specimens (Kim et al, 2011). Here, we found that CpG islands of miR-10b/10b* are highly methylated in some tumour specimens when compared to their matched peritumoural samples (Fig 2). The expression of miR-10b/10b* is regained when breast cancer cell lines are treated with demethylating agents (Fig 2). Growing evidence shows that miRs exert pleiotropic activities by controlling the expression of diverse target mRNAs. It has been shown that miR-10b suppresses the synthesis of Hoxd10 protein increasing the expression of the pro-metastatic gene products RhoC, urokinase activator plasminogen receptor (uPAR), a3-integrin and MT1-MMP (Ma et al, 2007; Sasayama et al, 2009; Sun et al, 2011). Other validated mRNA targets of miR-10b include KLF4 in human oesophageal cancer cell lines (Tian et al, 2010) and the nuclear receptor co-repressor 2 (NCOR2) in neuroblastomas (Foley et al, 2011). The computational analysis reveals that putative target genes of miR-10b* which are anti-correlated to the miR expression levels, are involved in various cellular pathways (Supporting Information Table S5A). Among those, seven putative target mRNAs encode for proteins involved in the control of cell cycle (Supporting Information Table S5B). Here, we document that down-regulation of miR-10b* correlates with aberrant expression of PLK1, BUB1 and CCNA2 proteins in breast cancer specimens when compared to their matched peritumoural samples. Restoration of miR-10b* expression by using mimic 10b* reduces PLK1, BUB1 and CCNA2 protein expression in diverse breast cancer cell lines (Fig 4E–G). The miR-10b* antagomiR expression increases the protein levels of PLK1, BUB1 and CCNA2 in MCF-10A cells (Fig 4H–J). siRNA-mediated down-regulation of PLK1 or BUB1 or CCNA2 impairs the ability of MCF-7 cells to form colonies (Supporting Information Fig S4J–S4L). miR-10b* targets the 3′-UTR region of *BUB1*, *PLK1* and *CCNA2* transcripts (Fig 4B–D). Interestingly, the analysis of gene expression databases of different cohorts of breast cancer patients reveals that those expressing high levels of PLK1, BUB1 or CCNA2 exhibit lower disease-free survival when compared to those expressing low levels (Fig 5A). High expression of BUB1 correlates with shorter relapse-free survival (Fig 5B). Patients with high expression of BUB1 and CCNA2 exhibit reduced metastasis-free survival when compared to patients with low expression (Fig 5C).

It has been reported that miR-10b antagomir suppresses breast cancer metastasis *in vitro* and *in vivo* without having any effect on the primary breast tumours (Ma et al, 2010). Here, we provide evidence that xenografted human breast tumours in mice, when injected with synthetic miR-10b*, show impaired tumour growth characterized by a significantly reduced proliferative index (Fig 6A–E).

Collectively, these findings have several implications: (a) miR-10b* down-regulation has a profound impact on breast cancer proliferation by disabling proper cell cycle regulation; (b) the expression of BUB1, PLK1 and CCNA2 proteins, whose respective transcripts are targets of miR-10b*, is dysregulated in different human cancers. This may suggest that alteration of miR-10b* expression plays a role in establishing also other types of tumours. Indeed, we found that down-regulation of miR-10b* occurs also in gastric and head and neck tumours when compared to their matched peritumoural tissues (Unpublished observation by FB, VC, AS, FG and GB); (c) the fine deciphering of the molecular events governing the expression of the miR-10b locus (miR-10b* and miR-10b) may disclose novel therapeutic targets to tackle breast tumourigenesis.

## MATERIALS AND METHODS

### Cell viability and colony-formation assay

A half millilitre aliquot of cell suspension (obtained from MCF7 and MDA_MB_468 cells transfected or not with miR-10b* oligos), was mixed with 0.5 ml of 0.4% trypan blue dye and left for 5 min at room temperature. The cells were counted using the Thoma chamber counting cells and the number of viable cells was determined. The experiment was conducted in triplicate and every point was counted 4 times. With regard to colony-forming assays, 1 × 10^3^ cells were seeded in 60-mm dishes and grown for 21 days. Cells were stained with crystal violet and colonies counted.

### Terminal deoxynucleotidyl transferase-mediated dUTP nick end labelling assay

The DeadEnd Fluorimetric [terminal deoxynucleotidyl transferase-mediated dUTP nick end labelling (TUNEL)] system from Promega Corp. (Madison, WI) was used to detect apoptosis by fluorescence-activated cell sorting analysis in the MDA-MB-468 cells following the manufacturer's protocol. Cellular fluorescence was measured using a Guava EasyCyte 8HT flowcytometer.

### Flow cytometry

Cells were harvested, washed with PBS and resuspended in 75% ethanol in PBS and kept at −20°C for at least 30 min. Cells were resuspended and incubated for 10 min in a solution containing 0.1% NP40 and 1 mg/ml RNAse A in PBS. Propidium iodide (Sigma) at a final concentration of 0.05 mg/ml was then added. The suspension was then analysed using a Guava EasyCyte 8HT flow cytometer. All flow cytometry data were analysed using the FlowJo software (Tree Star, Ashland, OR, USA).

### Patients and samples

One hundred and twenty pairs of primary breast cancers were obtained from patients from Italian National Cancer Institute ‘Regina Elena’. Matched peritumour non-cancerous breast tissues were also obtained from all the patients included in the study. The latter was approved by the scientific ethic committee from Italian National Cancer Institute ‘Regina Elena’ (protocol number 5E/459/10). Informed consent was obtained from all subjects. All experiments present in this work conformed to the principles set in the WMA Declaration of Helsinki and the NIH Belmont Report.

For the purpose of our study, only histologically uninvolved breast tissue sampled within 2 cm from tumour margin is defined as peritumoural tissue. All tissue samples used throughout the study were histologically examined before starting the experiments. Two additional cases of reductive mammoplasty (normal samples derived from individuals without cancer) were used as healthy control. The cohort for microarray analysis consisted of 56 matched breast tumour specimens, 5 unmatched tumour samples and 3 unmatched peritumour samples. Following excision, tissue samples were immediately frozen in liquid nitrogen and stored at −80° until RNA extraction. The cohort of paraffin-embedded tissues used for array validation consisted of 59 breast tumour specimens. Clinical and pathological data relating to the clinical samples are shown in Supporting Information Tables S1 and S4. ER, PRg and HER2/*neu* status of the patients were determined by IHC on formalin fixed, paraffin embedded sections of clinical specimens as part of routine pathology to guide clinical decision regarding adjuvant therapy. IHC was performed using rabbit monoclonal anti human ER antibody (Dako, UK) and a polyclonal rabbit anti human PRg antibody (Dako, UK). Membranous staining was scored for HER2/neu (Dako, UK).

### RNA extraction, labelling and microarray hybridization

Breast tumour tissue (50–100 mg) was manually homogenized in 1 ml of TRI Reagent lysis reagent (Ambion) according to manufacturer's instructions. The concentration and purity of total RNA were assessed using a NanodropTM 1000 spectrophotometer (Nanodrop Technologies, Wilmington, DE, USA). Total RNA (100 ng) was labelled and hybridized to Human miRNA Microarray V2 (Agilent). Scanning and image analysis were performed using the Agilent DNA Microarray Scanner (P/N G2565BA) equipped with extended dynamic range (XDR) software according to the Agilent miRNA Microarray System with miRNA Complete Labeling and Hyb Kit Protocol manual. Feature Extraction Software (Version 10.5) was used for data extraction from raw microarray image files using the miRNA_105_Dec08 FE protocol. miRNA expression data was deposited in Gene Expression Omnibus (GEO) with accession number GSE40525.

### Microarray data analysis

#### Normalization

All arrays were normalized together using a Lowess multi-array algorithm (Ballman et al, 2004), miRs that were not detected in all samples according to GeneView flags were removed and all values lower than 5 were considered below detection and thresholded to 5.

#### Noise estimation

Noise in the expression levels was estimated on the basis of 16 samples for which two replicates were measured. The noise did not depend on the miR intensity levels and therefore was calculated for all miRs together. Noise was estimated by the standard deviation of the intensity differences between replicate samples.

#### Comparison of matched sample pairs (CMSP)

For each patient with both tumour and matched peritumour sample, a *p*-value was calculated, for each miR, based on the difference of expression of the miR between the two matched samples, divided by the noise (standard deviation). The *p*-values for each specific miR from all patients (from the same subtype) were combined together into one *p*-value, using Fisher's method (Mosteller & Fisher, 1948). Thus, for each miR three *p*-values were calculated (one for each disease subtype).

#### Identifying differentially expressed miRs between tumour and peritumour samples in the various subtypes of breast cancer

Three statistical tests were used to define the group of differentially expressed miRs in each subtype: (i) paired *t*-test (ii) 2-samples *t*-test and (iii) CMSP (see above). For explanations and rationale for combining the three tests see Supporting Information. After performing these three tests, a combined *p*-value is calculated for each miR in each subtype, using Fisher's method. To define the group of differentiating miRs for each subtype, a version of the FDR procedure was used (Zeisel et al, 2011). The level of significance used as threshold was different for each subtype (Supporting Information Table S2), since the number of patients in each subtype was different (and we wanted to get differentiating lists of about the same sizes). In addition to the threshold on the combined *p*-value, thresholds on all three tests were also used since the combined *p*-value is very sensitive to extremely low *p*-values and we wanted to eliminate miRs which got extremely low *p*-value in one test only (see Supporting Information Table S3A–S3C, for lists of differentiating miR in the various subtypes and their *p*-values). Note that for the HER2^+^ subtype all patients had matched samples and therefore the two-samples *t*-test was not used.

**The paper explained**

PROBLEM:

Breast cancer is still the leading cause of cancer death in women worldwide. The aetiology of this type of cancer is complex and both genetic and environmental factors contribute to its complexity. Deciphering the molecular mechanisms involved in breast tumourigenesis has been the subject of extensive research in last years; yet unpredictable response and development of resistance to adjuvant therapies remain major challenges in the management of breast cancer patients.

RESULTS:

In the present study, we show that down-regulation of miR-10b* expression, through hypermethylation of its regulatory regions, occurs specifically in breast tumour specimens when compared to their matched peritumoural samples. We demonstrate that miR-10b* targets the expression of PLK1, BUB1 and CCNA2 cell cycle regulatory proteins. Importantly, the aberrant expression of these proteins exhibits prognostic value in breast cancer. We also provide evidence that injection of synthetic miR-10b* impaired tumour growth of xenografted human breast tumours.

IMPACT:

Since miR-10b* down-regulation is an alteration common to the three major breast cancer subtypes, it might represent a critical molecular event for breast cancer establishment. Thus, restoration of miR-10b* expression might hold therapeutic potential for breast cancer treatment.

#### Sorting points into neighbourhood (SPIN)

SPIN is an unsupervised method for sorting and visualization of multidimensional data (Tsafrir et al, 2005); it allows finding groups of miRs that display similar expression patterns over a range of samples (see Supporting Information).

#### Coupled miR and mRNA expression data

We used the publicly available dataset GSE19536 (Enerly et al, 2011) from the Gene Expression Omnibus repository (Barrett & Edgar, 2006; Edgar et al, 2002), which contains miR and mRNA expression data of 100 breast cancer tumours. We calculated the Pearson correlation and corresponding *p*-values between expression levels of miR-10b* and its 736 predicted target genes.

#### mRNA expression data

We used the publicly available data sets: GSE4922 (Ivshina et al, 2006), GSE3494 (Miller et al, 2005) and SE6532 (Loi et al, 2007). The mRNA expression data was measured using Affymetrix Human Genome U133A and B arrays and the preprocessing steps included summarization by the Affymetrix Expression Console and normalization of all arrays (from each data set) together, using our version of Lowess multi-array algorithm (Ballman et al, 2004).

#### Kaplan–Meier analysis

The patients were divided into three equal groups based on the tested mRNA expression levels (low, medium and high). The Kaplan–Meier analysis (Clark et al, 2003) was performed between the highest and lowest groups, to test for an association between the mRNA expression levels and the survival of the patients. The medium group was excluded to ensure difference in expression between the highest and lowest groups.

### Methylated DNA immunoprecipitation assay (MeDIP)

DNA was isolated by incubating cells overnight at 37°C in SDS/proteinase K digestion buffer (NaCl 300 mM, EDTA 25 mM, Tris pH 8 50 mM, SDS 2% e Prot K 0.2 µg/µl). DNA was extracted twice with phenol/chloroform followed by ethanol precipitation. Pellets were resuspended in TE plus 20 µg/µl RnaseA and the resulting DNA was quantified on a Nanodrop spectrophotometer. Immunoprecipitation of methylated DNA was prepared as (Weber et al, 2005); the antibody against 5-methyl-cytosine used for immunoprecipitation was from Abcam ab1884, San Diego, CA. Quantitative PCR (qPCR) reactions were carried out in triplicate on specific genomic regions using TaqMan Master Mix (Applied Biosystem). The list of probes (IDT) used for amplification on methylated region was summarized in Supporting Information Table S9. The resulting signals were normalized for primer efficiency by carrying out qPCR for each primer pair using Input DNA.

### Bisulphite sequencing

Genomic DNA was extract with Trizol protocol and purified with Wizard genomic DNA purification kit (Promega, Madison, WI). A total of 500 ng of genomic DNA was converted with Qiagen EpiTect Bisulphite kit following the manufacturer's instructions and amplified by PCR with primer set designed using MethPrimer software (Li & Dahiya, 2002). Primer sequences are presented in Supporting Information Table S10. PCR product were isolated from 2% agarose gels using Nucleospin extract II (Macherey Nagel). Subsequently, they were ligated into pCR2.1 using TA cloning kiy (Invitrogen). Individual clones were sequenced on Big Dye V3.1 Cycle-Sequencing kit (Applied Biosystems) with M13 Fw and M13 Rw primers. Sequencing reactions were analysed on to 3130 Genetic Analyzer (Applied Biosystems). Multiple clones (∼20) were sequenced and average methylation levels are represented. All clones had a C to T conversion at non-CpGs higher that 99%.

### Breast cancer xenografts

MDA-MB-468 control (siGFP) or triple siRNA (siBUB1, siPLK1 and siCCNA2) transfected cells were trypsinized, counted and subcutaneously injected into the lower back of 4-week-old SCID mice (Charles River Laboratories, Lecco, Italy) using 10 × 10^6^ cells in 100 µl DMEM with 30% matrigel (BD Biosciences, San Jose, CA, USA) per injection. Once cancer cells have developed palpable tumours, caliper measurements were taken and tumour volume was calculated using the formula {*V* = [length × (width)2]/2} (by manual caliper). For miR-10b* injection experiment, 50 µl synthetic miRNA (Austin, TX, USA; pre-miR, cat. no. AM17100) complexed with the siPORTamine transfection reagent (Ambion, Austin) was delivered intratumourally in 3-day intervals, when tumours reached an average volume of 100 mm^3^. For each injection, 6.25 µg miRNA was complexed with 1.6 µl siPORTamine (Ambion; cat. no. AM4502) reagent in 50 µl PBS. At the end of the treatments animals were sacrificed in accordance with standard protocols, tumours were collected and prepared for histology and RNA isolation. All the procedures involving animals and their care were approved by the Ethical Committee of Italian National Cancer Institute ‘Regina Elena’ and conformed to the relevant regulatory standards in accordance with Italian legislation.

For more detailed Materials and Methods see the Supporting Information.

## Supplementary material

Detailed facts of importance to specialist readers are published as ”Supporting Information”. Such documents are peer-reviewed, but not copy-edited or typeset. They are made available as submitted by the authors.
