# Supplementary material for: miR-10b*, a master inhibitor of the cell cycle, is down-regulated in human breast tumours
Source: EMBO Mol Med. 2012 Nov 2;4(11):1214–29. doi: 10.1002/emmm.201201483 (PMC3494877; doi:10.1002/emmm.201201483)
Supplement: Supplementary file 1 [file emmm0004-1214-SD1.pdf]

EMBO
Molecular Medicine

Manuscript EMM-2012-01483

miR-10b*, a master inhibitor of the cell cycle, is down-regulated in human breast tumors

Biagioni F, Bossel Ben-Moshe N, Fontemaggi G, Canu V, Mori F, Antoniani B, Di Benedetto A, Santoro R, Germoni S, De Angelis F, Cambria A, Avraham R, Grasso G, Strano S, Muti P, Mottolese M, Yarden Y, Domany E and Blandino G

Corresponding author: Giovanni Blandino, Italian National Cancer Institute 'Regina Elena'

Review timeline:

Submission date:	19 April 2012
Editorial Decision:	15 May 2012
Revision received:	10 August 2012
Editorial Decision:	24 August 2012
Revision received:	07 September 2012
Accepted:	10 September 2012

Transaction Report:

(Note: With the exception of the correction of typographical or spelling errors that could be a source of ambiguity, letters and reports are not edited. The original formatting of letters and referee reports may not be reflected in this compilation.)

1st Editorial Decision 15 May 2012

Thank you for the submission of your manuscript to EMBO Molecular Medicine. We have now heard back from the two referees whom we asked to evaluate your manuscript.

As you will see from the reports below, the referees find the study to be of potential interest. They do however raise a number of concerns. In particular, Referee 1 would like to see a better clarified message (point 1) and convincing target validation (point 4). The referee 2 is more concerned about technical issues and make constructive suggestions to improve the manuscript.

Given the balance of these evaluations, we feel that we can consider a revision of your manuscript if you can address the issues that have been raised within the space and time constraints outlined below. Please note that it is EMBO Molecular Medicine policy to allow only a single round of revision and that, as acceptance or rejection of the manuscript will depend on another round of review, your responses should be as complete as possible.

Revised manuscripts should be submitted within three months of a request for revision; they will otherwise be treated as new submissions, except under exceptional circumstances in which a short extension is obtained from the editor. Also, the length of the revised manuscript may not exceed 60,000 characters (including spaces) and, including figures, the paper must ultimately fit onto optimally ten pages of the journal. You may consider including any peripheral data (but not methods in their entirety) in the form of Supplementary information.

I look forward to seeing a revised form of your manuscript as soon as possible.

Yours sincerely,

Editor
EMBO Molecular Medicine

***** Reviewer's comments *****

Referee #1:

The authors identify a miRNA, miR-10b*, which is downregulated in breast tumor samples when compared to peritumoral tissue in a variety of breast tumor subtypes. miR-10b* downregulation is ascribed (mainly) to the hypermethylation of two CpG islands close to miR-10b/miR-10b* encoding locus. They then demonstrate that ectopic miR-10b* impairs the proliferative capacity of breast cancer cell lines in vitro (MCF7 and MDA MB 468). Moreover, in mouse models the growth of subcutaneous xenografts of MDA MB 468 cells is arrested by intratumoral inoculations of miR-10b*. In human breast tumor samples, an inverse correlation exists between miR-10b* levels and tumor size.
Given the apparent relationships between growth and miR-10b, the authors propose a role for miR-10b* in the regulation of cell cycle progression; they identify, among predicted miR-10b* target mRNAs, a list of genes involved in the cell cycle pathway. Three of such genes (BUB1, PLK1 and CCNA2) are validated in vitro as miR-10b* targets; BUB1, PLK1 and CCNA2 are shown to be highly expressed in a fraction of tumor samples and poorly expressed in the matched peritumoral tissue and to have a prognostic value in some patients' datasets.

This is very interesting work. They only need to address few remaining points:

Only one MAIN CONCERN:
1) The MCF7 data are state of the art and sufficient to support the mechanistic claims; This is not the case for MDA-468, as data here are more in favor of promotion of cell death rather than cell-cycle control. please clarify your key claim. See also point 5 below. Of course death may follow growth arrest; in other words this would not be a problem per se (I would be just fine with an "hybrid" mechanism of cell death + slowed cell cycle...) only that they dedicated 2 figures on kinases/proteins affecting cell cycle progression.

The rest are Minor issues, that may be addressed very easily.

2) - Fig 2G: why is peritumoral different than normal? The difference is stronger here than in the two experimental samples... What's so different? Please clarify. This is also ill defined in the methods (where is the peritumoral coming from?) How did they make sure that this is not contaminated from tumor stroma, inflammatory cells, or tumor cells?

3)
- Fig 3 ed S3: please show the cell cycle analyses, that is, the % of cells in the various phases (data in SI is very poor and too limited). As for TUNEL, they should count the number of positive cells in control vs miR-10b* gain.
-
-
4) Fig 4D,E on target validation: Westerns are fine. But the 3'-UTR are far less convincing (for CCNA2 and PLK1). I am wandering if there is a technical issue: the transfection was incomplete, the lux-accumulation is cutting short any difference (if so try to repeat the assay by leaving more time for the lux to decay).

5) Again on point 1. Functional data are on tumor size and Ki-67 on MDA468, where it is very clear that the effect of the miR is on cell death/apoptosis (see the sub-G1 accumulation). Please clarify why not using orthotopic transplantation.
The assay they use is very nice: they inject the miR after tumor growth. Is this affecting apoptosis (as they do in vitro) and/or necrosis?

6 Please clarify why the miRNA lists should be of the same "size"/number for different tumor subtypes?

7 Fig 4P: what is the loading control?

Discussion:

- I understand that miR10b should go down (by Methylation of the promoter) in primary tumors (and this may be a general phenomenon). However, in other primary tumors and their metastases miR-10b needs to be made competent again to be transcribed by Twist etc (Weinberg and colleagues, Ma et al., Nature 2007). Is this correct? The fact that in the present MS they also look at metastasis-free survival using the miR-targets is a bit confusing.

- the expression of BUB1, PLK1 and CCNA2 proteins, whose respective transcripts are targets of miR-10b*, is disregulated in different human cancers. This may suggest that alteration of miR-10b* expression plays a role in establishing also other types of tumors.
This statement is too broad. Please clarify.

Referee #2:

The manuscript by Biagioni et al. shows that miR-10b* is downregulated in human breast cancer due to hypermethylation of the CpG islands upstream of the mir-10b/10b* locus. Ectopic expression of miR-10b* inhibited proliferation and colony formation, and promoted apoptosis of human breast cancer cells in vitro. The authors indentified three miR-10b* target genes involved in cell cycle regulation (BUB1, PLK1 and CCNA2) and showed that high expression levels of these target genes correlated with poor prognosis in breast cancer patients. Intratumoral injection of miR-10b* mimics significantly suppressed in vivo tumor growth, which suggested potential therapeutic application of miR-10b*.

Specific points:

1. Figure 2B: a specificity control for the pull down with the 5-methyl-cytosine antibody is needed here.

2. Figure 4I-M: the efficiency of the miR-10b* antagomirs should be shown: if these antagomirs can degrade miR-10b*, then qPCR analysis of miR-10b* should be shown; otherwise, the authors should determine the functional knockdown efficiency by using a reporter assay.

3. Figure 4O, P: it is important for the authors to exclude the off-target effect of siRNA (which is a common standard for publishing siRNA results nowadays); this can be done by including additional siRNAs (not pooled), or better, by doing a rescue experiment with a construct that does not bind to the siRNA used.

4. The selection of BUB1, PLK1, and CCNA2 as the key functional targets of miR-10b* in tumorigenesis (actually, even for these three selected targets, no in vivo data are shown - see point #5 below) appears arbitrary. To use an unbiased approach to determine the mechanisms by which miR-10b* suppresses tumorigenesis, the authors should consider performing microarray (or RNA-seq) analysis of miR-10b*-expressing tumor cells.

5. More evidence is required to demonstrate that the in vivo tumor-suppressing effect of miR-10b* depends on the inhibition of the three candidate targets identified: 1) does knockdown of the three targets recapitulate the tumor-suppressing effect of miR-10b* in vivo? 2) Conversely, does restoration of the three targets in miR-10b*-overexpressing tumor cells reverse the effect of miR-10b* on tumorigenesis in vivo?

6. Figure 5: the authors show that expression of the three miR-10b* targets correlates with poor prognosis, but do not show whether expression of miR-10b* correlates with clinical outcomes.

7. Figure 6E: the difference in the percentage of Ki-67+ cells is moderate (P = 0.044). The authors should examine other proliferative markers as well as apoptotic markers.

1st Revision - Authors' Response 10 August 2012

Referee #1

We appreciate very much that the reviewer has found our work very interesting. We thank the reviewer for the helpful suggestions for which we provide a point by point response.

This is very interesting work. They only need to address few remaining points:

Only one MAIN CONCERN:

1) The MCF7 data are state of the art and sufficient to support the mechanistic claims; This is not the case for MDA-468, as data here are more in favour of promotion of cell death rather than cell-cycle control. please clarify your key claim. See also point 5 below. Of course death may follow growth arrest; in other words this would not be a problem per se (I would be just fine with an "hybrid" mechanism of cell death + slowed cell cycle...) only that they dedicated 2 figures on kinases/proteins affecting cell cycle progression.

To address the concern regarding MDA-MB-468 cells we have performed three distinct sets of experiments:

A) As reported in the new fig 3E, we have analysed cell cycle profile of MDA-MB-468 cells upon overexpression of miR-10b*. We found that at 72h the increase of subG1 and related decrease of G1 was statistically significant (subG1 p value = 0.04, G1 p value = 0.02). We have been unable to identify a time-point along which we could find evidence for an increase of G1 fraction of the cells which precedes what we found in subG1. This might suggest that the pool of subG1 cells originates from G1 fraction and represents a specific feature of MDA-MB-468 when compared to other cell lines as MCF7 cells.

B) As the reviewer suggested in point 5 which is closely connected to point 1, we have deeply analysed the tumors whose volume was severely reduced by the injection of miR-10b*. As reported in the new fig 6D-E, we now provide evidence of a clear reduction of ki67 in all 5 representative mice analysed. We also stained these sections for TUNEL positivity and we evidenced a statistically significant (p value = $6{\cdot}10^{-5}$) difference between control mice and miR-10b* injected mice (2% versus 5%, new Supporting Information Figure 6A). This again suggests that the major effect of miR-10b* occurs at the level of inhibition of proliferation as evidenced by ki67 and cyclin D1 analysis (new figure 6D), which is accompanied by a small degree of apoptosis (new Supporting Information Figure 6A). Notably, the expression of BUB1, PLK1 and CCNA2 are significantly (p value BUB1 = $7.5{\cdot}10^{-8}$, p value PLK1 = 0.0015, p value CCNA2 = 0.005) reduced in tumours injected with miR-10b* (figure 6D-E).

C) As reported in the new Figures 4O-P, MDA-MB-468 cells whose endogenous expression of PLK1, BUB1 and CCNA2 was selectively knocked down, engrafted less efficiently than control cells. This is again accompanied by a reduction of proliferative index, as shown by the reported ki67 analysis (new Figure 4P).

The rest are Minor issues, that may be addressed very easily.
2) Fig 2G: why is peritumoral different than normal? The difference is stronger here than in the two experimental samples... What's so different? Please clarify. This is also ill defined in the methods (where is the peritumoral coming from?) How did they make sure that this is not contaminated from tumour stroma, inflammatory cells, or tumour cells?

Only glandular breast epithelium sampled from non cancer-containing breast, in the absence of morphological alterations, should be considered as normal breast. Peritumoral breast glandular epithelium, in fact, according to the field cancerization hypothesis (Braakhuis et al., 2003), could harbour molecular changes heralding early stages of cancer development (Botti et al., 2000; Braakhuis et al., 2003). This may suggest that some tumour perturbations may occur in breast tissue before morphological changes are apparent. In this context, peritumoral tissues should be considered different from normal tissues. In our series, we found that miR-10b* is down regulated in breast cancer samples when compared to uninvolved peritumoral tissues, independently of breast tumour subtypes. Comparison of peritumoral and normal tissues demonstrated that a certain percentage of miR-10b* locus methylation is present also in peritumour, as compared to normal tissues. This could rely on molecular alterations present in peritumour tissues of breast cancer patients and not in healthy donors (normal samples are indeed derived from reductive mammoplasty of individuals without cancer).
For the purpose of our study only histologically uninvolved breast tissue sampled within 2 cm from tumour margin is defined as peritumoral tissue. We added this definition in Materials and Methods section (page 13-14).
All tissue samples used throughout the study were histologically examined before starting the experiments. Furthermore for FFPE tissues before extracting miRNAs, we were careful in selecting only glandular areas (avoiding stromal contamination as much as possible) from the section. Modulation of the 5 differentially modulated miRs was similar between frozen and FFPE samples.

3) Fig 3 ed S3: please show the cell cycle analyses, that is, the % of cells in the various phases (data in SI is very poor and too limited).
As for TUNEL, they should count the number of positive cells in control vs miR-10b gain.*

We now include percentage of the cells in the various phase of cell cycle in Fig. 3E and Fig. 3H.

We apologize with the reviewer as we made a mistake in labelling the Y axis of the histogram shown in Fig. 3G. The histogram shows the percentage of TUNEL positive cells, therefore we changed the labelling accordingly. In addition, we are providing a histogram showing the mean fluorescence intensity for the three samples (new Supporting Information Fig. S3A). As the reviewer can see, the data for both mean fluorescence and percentage of TUNEL positive cells vary accordingly.

4) Fig 4D,E on target validation: Westerns are fine. But the 3'-UTR are far less convincing (for CCNA2 and PLK1). I am wandering if there is a technical issue: the transfection was incomplete, the lux-accumulation is cutting short any difference (if so try to repeat the assay by leaving more time for the lux to decay).

According to the reviewer's suggestion, we modified luciferase reading conditions and performed a new set of transfections. New data are shown in the new fig. 4 B-D.

5) Again on point 1. Functional data are on tumour size and Ki-67 on MDA468, where it is very clear that the effect of the miR is on cell death/apoptosis (see the sub-G1 accumulation). Please clarify why not using orthotopic transplantation. The assay they use is very nice: they inject the miR after tumour growth. Is this affecting apoptosis (as they do in vitro) and/or necrosis?

See the answer to point 1. We agree with the reviewer that the use of orthotopic transplantation would have been more relevant for elucidating the therapeutic implications of our findings. Orthotopic transplantation will be performed in a planned follow-up study, in which our main aim will focus on the therapeutical relevance of miR-10b*.

6 Please clarify why the miRNA lists should be of the same "size"/number for different tumour subtypes?

© EMBO

In fact the reviewer is right – there is no reason to assume that our lists should contain the same number of miRs for different tumour subtypes. With this said, we think that it is reasonable to assume that the numbers of miRs that are activated and involved in the various subtypes of breast cancer are of the same order of magnitude (i.e. not hundreds for one subtype and less than ten for another). There is no evidence that indicates that for one specific subtype miRs play a crucial role while in another subtype they are negligible.
It so happens that our collection of samples contains much more Luminal tumours (reflecting the situation in the population), and therefore the tests for statistical significance yielded much lower p-values for this subtype; had we used the same threshold FDR levels for all subtypes, the Luminal list would have contained much more miRs (hundreds) versus the other subtypes (which would have yielded few tens). In order to eliminate this artificial effect, we used different FDR levels for each subtype to get about the same numbers of miRs on the lists for different subtypes.

7 Fig 4P: what is the loading control?

The loading control was assessed as GAPDH expression. We are sorry to have forgotten to indicate in the previous figure. Please find the figure complete (new Supporting Information Figure 4G).

Discussion:

- I understand that miR10b should go down (by Methylation of the promoter) in primary tumours (and this may be a general phenomenon).
However, in other primary tumours and their metastases miR-10b needs to be made competent again to be transcribed by Twist etc. (Weinberg and colleagues, Ma et al., Nature 2007). Is this correct? The fact that in the present MS they also look at metastasis-free survival using the miR-targets is a bit confusing.

In our study we performed microarray analysis on *early* primary breast tumours and we showed that miR-10b and miR-10b* expression levels are down regulated compared to peritumoral matched tissue (Supporting Information Fig. S2A). The down regulation of miR-10b in primary tumours compared to normal tissue, was also demonstrated by Weinberg et al. 2007 and Iorio et al. 2005. According to Weinberg's paper, activation of Twist and the resulting induction of miR-10b expression occurs at *later* stages of primary tumour progression, indicative of its role in the invasion–metastasis transformation. Hence there is no contradiction with Weinberg (we look at tumors at different stages of progression). Furthermore, we claim that there is an association of miR-10b* levels with proliferation rate: high levels of miR-10b* correspond to lower proliferation. The biological processes governing proliferation and metastasis are different – it could be that low levels of miR-10b* expression contribute to proliferation (at an early stage) while high levels of miR-10b give rise to metastasis (at late stage).
Moreover, it is known that miR-10b up regulation is a typical event that occurs not only in metastatic breast tumours but also in very advanced tumours and in pancreatic adenocarcinomas (Bloomston et al. 2007 and Preis et al. 2011) and glioblastoma tumours (Ciafre et al. 2005), two types of highly metastatic and/or invasive cancers (see page 12 in the revised manuscript).
In order to check the association between miR-10b* and survival, we performed Kaplan-Meier analysis (Figure 5) on the three target genes of miR-10b* in three large datasets of primary breast tumors (Ivshina et al, Miller et al and Loi et al). We found that low expression levels of the 3 targets of miR-10b* are associated with better prognosis in terms of Disease free survival, Relapse free survival and Metastasis free survival. Since the analysis of Metastasis free survival was performed on early primary tumours (Loi et al.), i.e. before the induction of miR-10b by Twist, the results are not in contrast with the pro-metastatic role of miR-10b, that can be reactivated in later stages of the tumour.

- the expression of BUB1, PLK1 and CCNA2 proteins, whose respective transcripts are targets of miR-10b, is disregulated in different human cancers. This may suggest that alteration of miR-10b* expression plays a role in establishing also other types of tumours.*
This statement is too broad. Please clarify.

We have added a sentence in the discussion section in which we report our unpublished observation

that miR-10b* down regulation occurs also in gastric and head and neck tumours when compared to their matched peritumoral tissue.

Referee #2

We appreciated very much reviewer's comment aimed at improving the in vivo relevance of our findings. We provide a point by point response to each of the raised specific comments.

Specific points

1.Figure 2B: a specificity control for the pull down with the 5-methyl-cytosine antibody is needed here.

For the MeDIP experiment, genomic DNA was immunoprecipitated with 5-methyl cytosine antibody. As control we used chromatin immunoprecipitated with NoAb. We performed real time PCR analysis with specific probes amplifying CpG island #1 and #2 (see new Supporting Information Table 8). As control of the specificity we amplified a CpG free region located on chromosome 3.
The quantification of the experiments was performed applying the following formula:
% (5mC/INPUT-NoAb/INPUT). This formula was used for CpG island #1 and #2 probes, and for CpG free region primers. Relative enrichments shown in figure 2B represent ratios between % of methylation obtained in CpG island #1 (or #2) and CpG free region. The mean of three independent experiments was shown.

2. Figure 4I-M: the efficiency of the miR-10b antagomirs should be shown: if these antagomirs can degrade miR-10b*, then qPCR analysis of miR-10b* should be shown; otherwise, the authors should determine the functional knockdown efficiency by using a reporter assay.*

Real time PCR analysis performed in MCF10a cells transfected with antagomiR 10b* revealed a decrease (40% at 48h and 50% at 72h after transfection) of miR-10b* expression when compared with cells transfected with antagomiR control. These data are now shown in the new Supporting Information Fig S4E-F.

3.Figure 4O, P: it is important for the authors to exclude the off-target effect of siRNA (which is a common standard for publishing siRNA results nowadays); this can be done by including additional siRNAs (not pooled), or better, by doing a rescue experiment with a construct that does not bind to the siRNA used.

To address the reviewer's concern, we tested the efficiency of two additional siRNAs to knockdown the expression of each of the three miR-10b* targets of interest (BUB1, PLK1 and CCNA2). As shown in the new Supporting Figures S4H-I, for each target, the two additional siRNAs also down regulated the expression of the corresponding transcript. They were also capable to inhibit the ability of MCF7 to form colonies, as shown in the new Supporting Figure 4M-N.

4. The selection of BUB1, PLK1, and CCNA2 as the key functional targets of miR-10b in tumorigenesis (actually, even for these three selected targets, no in vivo data are shown - see point #5 below) appears arbitrary. To use an unbiased approach to determine the mechanisms by which miR-10b* suppresses tumorigenesis, the authors should consider performing microarray (or RNA-seq) analysis of miR-10b*- expressing tumour cells.*

To address the reviewer's concern we now provide a detailed explanation of how these three targets were selected.
First, we extracted all predicted target genes of miR-10b* from Microcosm Targets (the only miR target prediction algorithm which contained predictions for miR-10b*). Since sequence-based target prediction algorithms are known to produce many false positive targets, we now used correlation of expression of the miR with its putative targets as a filter. Using the Enerly et al. dataset, which contains both mRNA and miR expression measurements from breast cancer primary tumours, we calculated the correlation between miR-10b* expression levels and those of its 736 predicted target

genes. 42 of the predicted target genes were significantly (10% FDR) anti-correlated with the miR expression levels, and we identified these as our candidate targets. Using now the DAVID tool, we found that these 42 genes were significantly enriched for the cell cycle pathway (with 7 out of 42 genes, corresponding to <0.001% FDR) – much more than any other pathway (see Table below).

KEGG pathway	#genes	p-value	FDR (%)
Cell cycle	7	$3.8 \cdot 10^{-8}$	$2.6 \cdot 10^{-5}$
Progesterone-mediated oocyte maturation	4	$5.1 \cdot 10^{-4}$	0.35
p53 signalling pathway	3	0.007	4.92
Oocyte meiosis	3	0.019	11.99

Thus, cell cycle is the most promising candidate pathway to be regulated by miR10b*.
Next, we checked the expression levels of *all* 7 target genes contained in the cell cycle pathway; among these, the three with the most negatively correlated expression to the miR were CCNA2, PLK1 and BUB1. Nevertheless, we did measure (by quantitative real time PCR) the expression levels of all 7 target genes in MCF7 cells with mimic control versus mimic miR-10b*; PLK1, CCNA2, CHEK1 and BUB1 were the genes whose expression levels were significantly reduced due to mimic miR-10b*. Therefore we decided to focus on these 3 candidate genes. Thus our selection was far from being "arbitrary". This process of selection is now explained in detail in the paper (pg.8).
We consider performing microarray or RNA-seq of miR-10b* overexpressing cells as further development of this work.

5. More evidence is required to demonstrate that the in vivo tumour-suppressing effect of miR-10b depends on the inhibition of the three candidate targets identified: 1) does knockdown of the three targets recapitulate the tumour-suppressing effect of miR-10b* in vivo? 2) Conversely, does restoration of the three targets in miR-10b*-overexpressing tumour cells reverse the effect of miR-10b* on tumorigenesis in vivo?*

1) To provide further evidence for the roles of BUB1, PLK1 and CCNA2 inhibition in the tumour suppressing effects of miR-10b*, we subcutaneously inoculated immunodeficient SCID mice with human MDA-MB-468 breast cancer cells transfected with specific siRNA molecules for BUB1, PLK1 and CCNA2. Before the inoculation, the transduced cells were checked for the efficiency of the silencing of BUB1, PLK1 and CCNA2 as shown in the new Figure 4N. Inoculated mice were sacrificed three weeks after cell inoculation and, as shown in the new Figure 4O, cells whose expression of BUB1, PLK1 and CCNA2 was concomitantly silenced, engrafted less efficiently than those transfected with control siRNA.

2) We have also attempted to address the second suggestion provided by the reviewer. We were unable to perform it due to the very low rate of cell clones overexpressing miR-10b*. This was expected as a consequence of the inhibition of cell proliferation induced by miR-10b* overexpression.

6. Figure 5: the authors show that expression of the three miR-10b targets correlates with poor prognosis, but do not show whether expression of miR-10b* correlates with clinical outcomes.*

We performed Kaplan-Meier analysis to evaluate the prognostic power of miR-10b* in breast cancer patients using the Enerly et al. dataset. Enerly et al. measured the expression levels of miR-10b* (by microarrays) in 78 primary breast tumours, for which survival information was available. We found that high expression levels of miR-10b* were associated with better prognosis, with a p-value of 0.156 (new Supporting Information Figure 5A). Although such a p-value is usually not considered significant, our analysis can be viewed as indicating a trend in the right direction. It should be born in mind that the relatively high p-value could be due to the number of samples, which is too low to yield reliable Kaplan – Meier analysis. Moreover, since miR-10b* expression levels are significantly anti-correlated with BUB1, PLK1 and CCNA2 expression levels (2% FDR), and low expression levels of these genes are significantly associated with better prognosis (based on 3 larger datasets, see Figure 5 in the paper), we can assume that high expression levels of miR-10b* would have been significantly associated with better prognosis in a larger dataset. An indirect evidence for

this can be obtained by analysis of the Buffa et al. dataset (Cancer Research 2011) of 210 breast tumours. Here the expression levels of miR-10b (but not of miR-10b*) were measured. Since miR-10b and miR-10b* are co-transcribed, the measured amount of miR-10b is indicative of the expression levels of miR-10b*. Indeed, Kaplan-Meier analysis of the samples, divided on the basis of miR-10b expression, yielded significant association with prognosis (p=0.0003, unpublished).

7. Figure 6E: the difference in the percentage of Ki-67+ cells is moderate (P = 0.044). The authors should examine other proliferative markers as well as apoptotic markers

To address the concern raised by the reviewer, we now provide the following evidence, presented in the new Fig 6D-E and in the new Supporting Information Figure 6A:

A) As additional marker of proliferation we analysed Cyclin D1 expression by immunostaining. As shown in the new Fig 6D-E (IV), the expression of Cyclin D1 was significantly (p value = 0.0008) reduced in tumours inoculated with miR-10b* mimic when compared to those inoculated with control mimic.
We also found a statistically significant (p value = 0.009) decrease in Ki67 expression (new Fig 6D-E (V)).

B) We also stained the tumour section used in the experiments described in point (A) for TUNEL positivity. We found a statistically significant (p value = $6 \cdot 10^{-5}$) difference between control mice and miR-10b* injected mice, as shown in the new Supporting Figure 5A.

C) We also stained the tumours used in points (A) and (B) to measure BUB1, PLK1 and CCNA2 protein levels. Of note, we found that the expression of all three proteins was significantly (p value BUB1 = $7.5 \cdot 10^{-8}$, p value PLK1 = 0.0015, p value CCNA2 = 0,005) reduced in tumours injected with miR-10b* compared to control (new Fig 6D-E).

All the data reported in the new Fig 6D-E were obtained by analysing five representative mice out of seven employed in the experiment

Altogether these data indicate that, at least under our experimental conditions, the in vivo tumour suppressor effect of miR-10b* occurs mainly through the inhibition of tumour cell proliferation which is accompanied by moderate apoptosis.

2nd Editorial Decision 24 August 2012

Thank you for the submission of your revised manuscript "miR-10b*, a master inhibitor of the cell cycle, is downregulated in human breast tumors" to EMBO Molecular Medicine. We have now received the reports from the reviewers who were asked to re-review your manuscript.

You will be glad to see that the reviewers are now globally supportive and we can proceed with official acceptance of your manuscript pending the minor changes detailed below:

- For experiments involving human subjects the submission must include a statement that informed consent was obtained from all subjects and that the experiments conformed to the principles set out in the WMA Declaration of Helsinki [http://www.wma.net/en/30publications/10policies/b3/] and the NIH Belmont Report [http://ohsr.od.nih.gov/guidelines/belmont.html]. Please see our Guide to Authors for further information and provide the necessary information in the respective Material and Methods part.

- Data of gene expression experiments described in submitted manuscripts should be deposited in a MIAME-compliant format with one of the public databases. We would therefore ask you to submit your microarray data to the ArrayExpress database maintained by the European Bioinformatics Institute for example. ArrayExpress allows authors to submit their data to a confidential section of the database, where they can be put on hold until the time of publication of the corresponding manuscript. Please see http:www.ebi.ac.uk/arrayexpress/Submissions/ or contact the support team at

arrayexpress@ebi.ac.uk for further information. Please include the accession numbers into an appropriate place in the manuscript.

- In addition, we noted that the resolution of some panels in Figure 4G (GAPDH) is rather low. Please include higher resolution pictures.

Please submit your revised manuscript within two weeks. I look forward to seeing a revised form of your manuscript as soon as possible.

Yours sincerely,

Editor
EMBO Molecular Medicine

***** Reviewers' comments *****

Referee #1:

I was happy with the MS in the first place and completely satisfied now. I think this is great and should be published..

Referee #2:

The authors have addressed my previous concerns.

2nd Revision - Authors' Response 07 September 2012

To address your requests, we made on the manuscript the following changes:

- For experiments involving human subjects we included in Materials and Methods section a statement of Italian National Cancer Institute 'Regina Elena' ethical committee stating that informed consent was obtained from all subjects
- We deposited on Gene Expression Omnibus (GEO) microRNAs expression profile data (GSE40525).
- For western blot in figure 4G, we acquired new images with better resolution for both CCNA2 and GAPDH in MCF7 and SKBR3 panel.
