# Supplementary material for: miR-10b*, a master inhibitor of the cell cycle, is down-regulated in human breast tumours
Source: EMBO Mol Med. 2012 Nov 2;4(11):1214–29. doi: 10.1002/emmm.201201483 (PMC3494877; doi:10.1002/emmm.201201483)
Supplement: Supplementary file 2 [file emmm0004-1214-SD2.pdf]

Supporting Information
Table of Content

The PDF file of Supporting Information is composed as follow:

- Supporting Information Figures and relative Figure legends
- Supporting Information Tables and relative Table legends
- Supporting Information Materials and Methods and References for this part

Supporting Information Figure S1 Biagioni et al

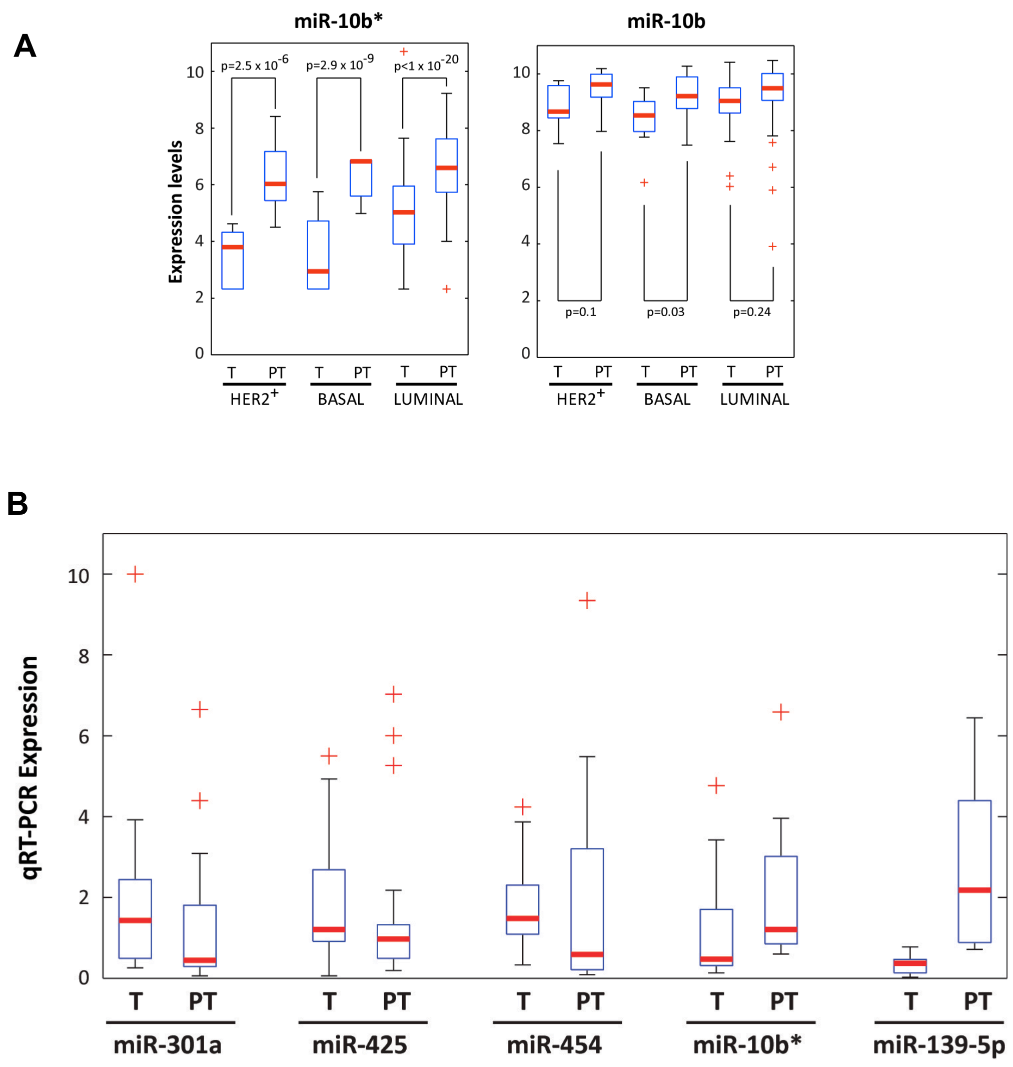

Supporting Information Figure S1

(A) Expression levels (log base 2 scale) of miR-10b* and miR-10b in tumor (T) and peritumor (PT) tissues of the various subtypes of breast cancer (HER2⁺, Basal-like and Luminal) from microarray data. (B) Expression levels of the five shared miRs: miR-301a, miR-425, miR-454, miR-10b* and miR-139-5, in tumor (T) and peritumor (PT) samples, measured by quantitative real time PCR in 12/18/12/12/8 representative matched samples, respectively. On each box, the central red mark is the median, the blue edges of the box are the 25th and 75th percentiles, the black lines extend to the most extreme data points not considered outliers, and outliers are plotted individually as +.

Supporting Information Figure S2 Biagioni et al

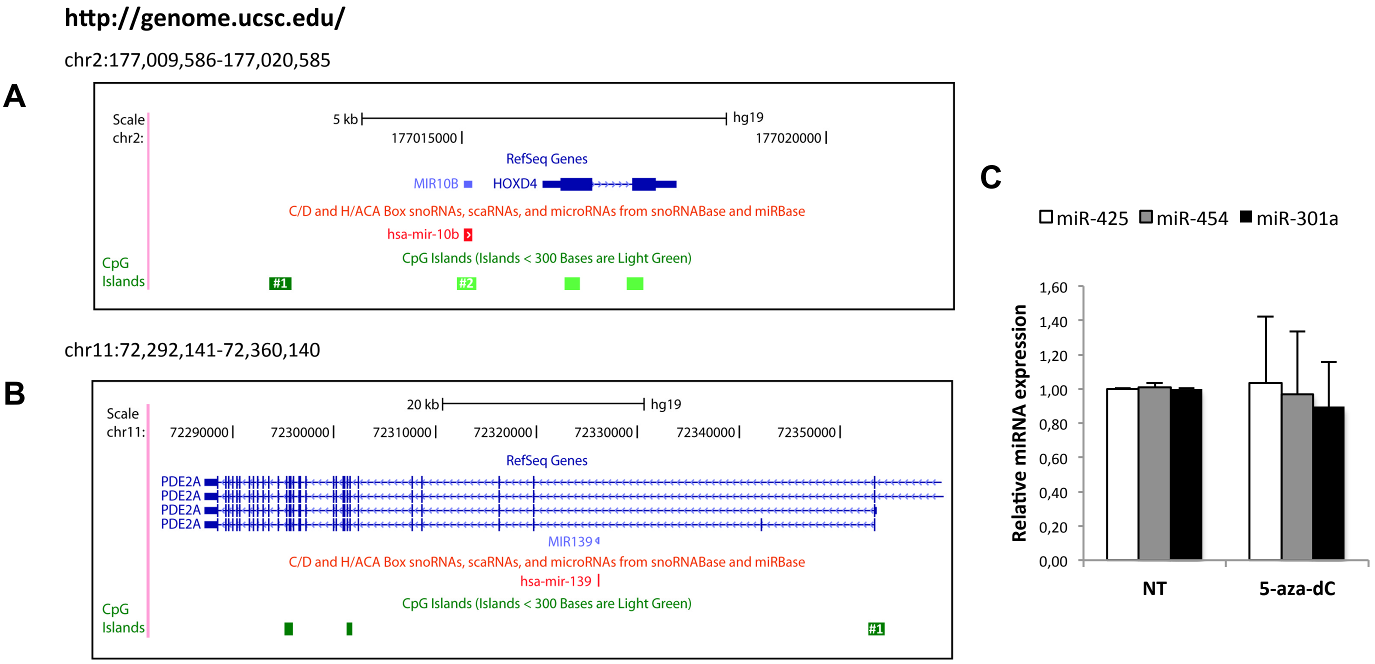

Supporting Information Figure S2

(A) Schematic representation of the microRNA-10b locus and (B) microRNA-139-5p locus and the CpG islands analyzed. (C) Analysis of miR-425, miR-454 and miR-301a expression after treatment with 5' aza dC.

Supporting Information Figure S3 Biagioni et al

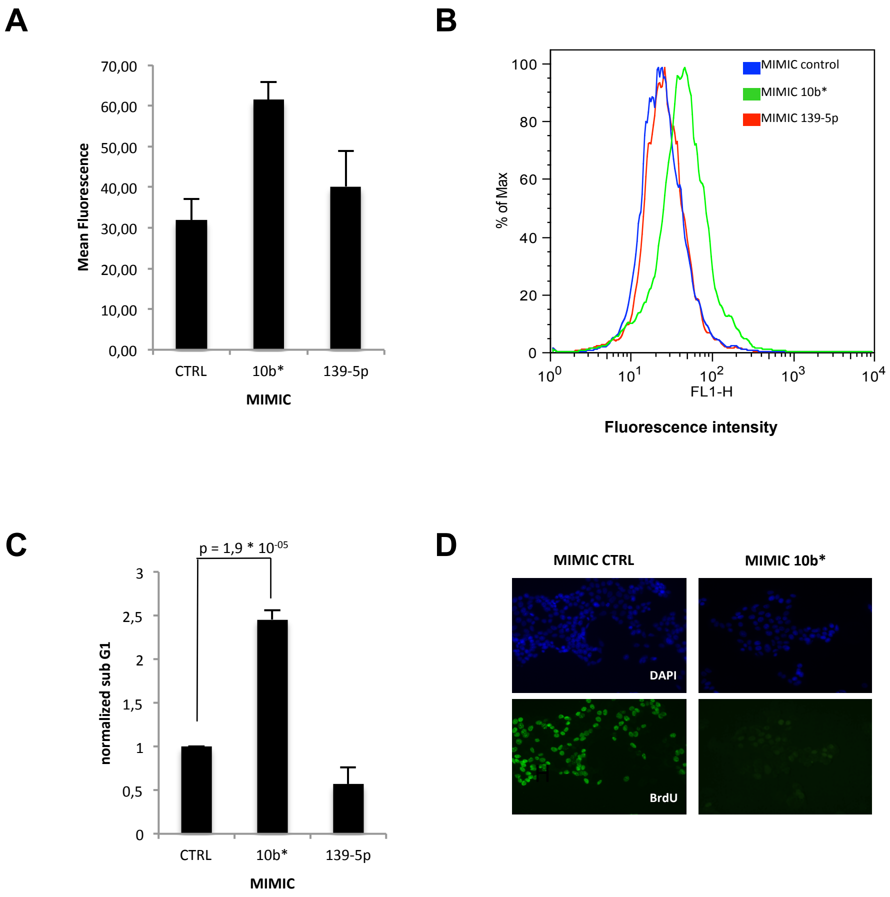

Supporting Information Figure S3

(A) TUNEL assay of MDA-MB-468 cells transfected with the indicated mimic molecules: mean fluorescence is shown. (B) Detection of apoptosis in MDA-MB-468 over-expressing mimic 10b* or mimic 139-5p by TUNEL assay: the *x* axis represents fluorescence intensity of DNA, and *y* axis represents the number of cells. (C) Histogram of three independent experiments showing normalizing sub G1 phase in MCF7 cells. (D) Incorporation of BrdU was detected by immunofluorescence in miR-10b* mimic or control MCF7 cells using monoclonal antibody against BrdU (bottom panels). Nuclei were counterstained with DAPI (blue).

Supporting Information Figure S4 Biagioni et al

A

Target gene	Correlation	P-value
CCNA2	-0,37	0,0001
PLK1	-0,36	0,0002
BUB1	-0,35	0,0003
CHEK1	-0,34	0,0005
CHEK2	-0,31	0,0015
CCNB3	-0,28	0,0045
CUL1	-0,26	0,0083

B

```
3' uaagGGGAUCUUA-GCUUAGACa 5' hsa-miR-10b*
       |: || ||| :|||||||
5' cacaCUGUA-AAUAUGAAUCUGc 3' BUB1 3'UTR

3’ UAAGGGGAUCUU-AGCUUAGACA 5’ hsa-miR-10b*
   |||:|: | ||| |||:| ||||
5’ ATTTCTATTGAATTCGGAACTGT 3’ PLK1 3’UTR

3’ TAAGGGGATCTTAGCTTAGACA 5’ hsa-miR-10b*
     ||||||     || | |||
5’  CTCCCCTC----CGGA-CTGG 3’ PLK1 3’UTR

3’ UAAGGGGAUCUUA-GCUUAGACA 5’ hsa-miR-10b*
   || :|::| | || :| ||||||
5’ ATATCTTTTGTATATGTATCTGT 3’ CCNA2 3’UTR
```

C

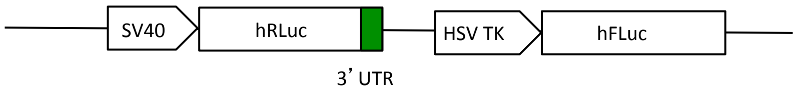

D

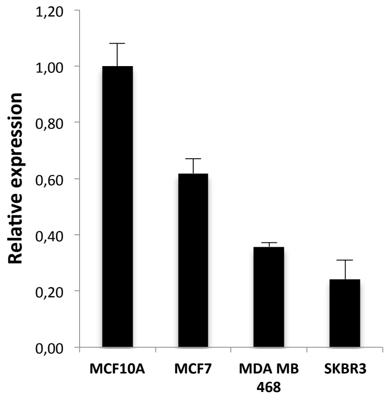

E

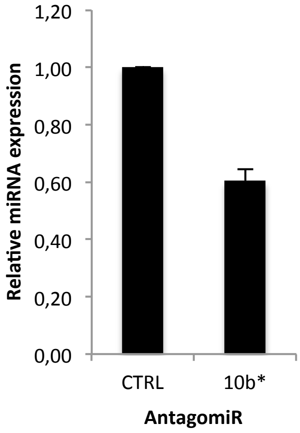

F

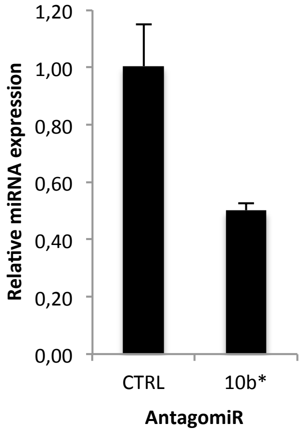

Supporting Information Figure S4 Biagioni et al

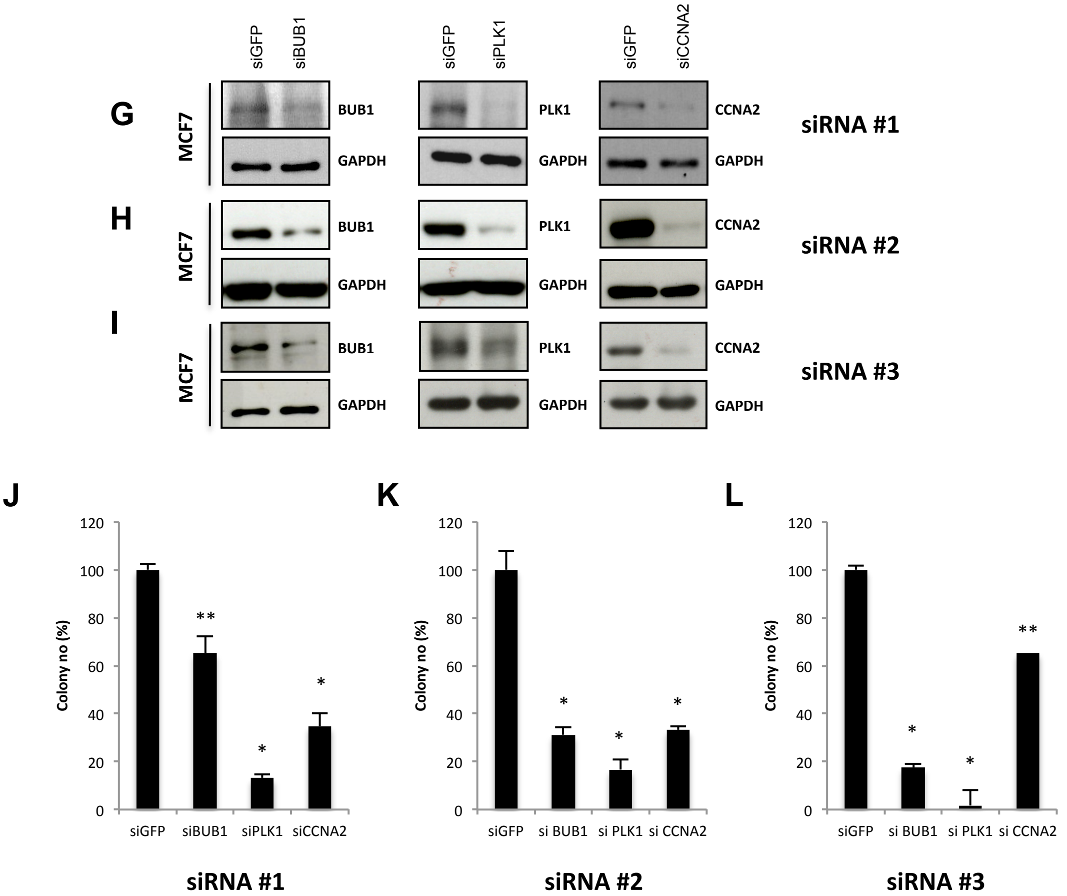

Supporting Information Figure S4

(A) The Pearson correlation coefficients and corresponding p-values between expression levels of miR-10b* and its seven predicted target genes in the publicly available dataset GSE19536 (Enerly et al). (B) The human BUB1, PLK1 and CCNA2 3-UTR harbor putative binding sites for miR-10b* (www.microrna.org). (C) Schematic representation of the luciferase reporter constructs used for transactivation assays. (D) Real time PCR of miR-10b* in four different mammal epithelial cells. (E-F) Real-time PCR of miR-10b* in cultured MCF10a cells treated with antagomiR control or antagomir-10b* at 48h (E) or 72h (F) from transfection. (G-I) Western Blot analysis showing the silencing of BUB1, PLK1 and CCNA2 expression in MCF7 cells with silencing #1 (G), silencing #2 (H) and silencing #3 (I). (J-L) Colony assay of MCF7 cells transfected with siGFP, siBUB1, siPLK1 or siCCNA2 with silencing #1 (J), silencing #2 (K) and silencing #3 (L). * indicates p < 0,005; ** indicates p < 0,05. An average of three independent experiments is reported. Colonies obtained in siGFP sample were set to 100%.

Supporting Information Figure S5 Biagioni et al

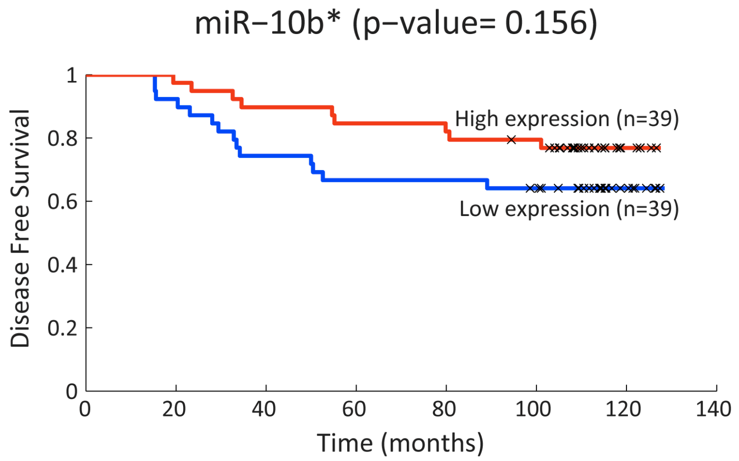

Supporting Information Figure S5

The association between the expression levels of miR-10b* and disease free survival was evaluated by Kaplan-Meier analysis in Enerly et al. datasets (78 primary breast tumors). The two compared groups are the half of patients with the highest expression levels of miR-10b* (red; n=39) versus the half of patients with the lowest expression (blue; n=39).

Supporting Information Figure S6 Biagioni et al

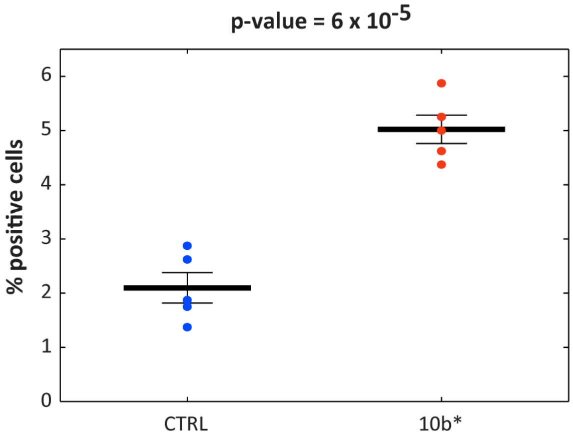

Supporting Information Figure S6

Immunohistochemistry quantification of TUNEL from five representative mice are shown. P-values were calculated by 2 sample t-test, and indicated in the figure.

Supporting Information Table S1 Biagioni et al

	N	%
**Number of patients**	61	
**Hystotype**		
Infiltrating Ductal Carcinoma	56	91,8
Infiltrating Lobular Carcinoma	5	8,2
**Grading**		
G1	3	4,9
G2	31	50,8
G3	27	44,3
**Tumor size**		
T1	17	27,8
T2	42	68,9
T3	2	3,3
**Nodal status**		
N0	29	47,5
N+	32	52,5
**Estrogen receptor**		
Positive (>10%)	47	77
Negative	14	23
**Progesteron receptor**		
Positive (>10%)	34	55,7
Negative	27	44,3
**HER2**		
Negative (score 0/1+)	41	67,2
Negative (score 2+ Not Amplified)	12	19,7
Positive (score 2+ Amplified + 3+)	8	13,1

Supporting Information Table S1

Clinical characteristics of study population and tumors used for microRNA expression profiles

Supporting Information Table S2 Biagioni et al

	# Samples			Significance level (FDR q-value)				Differentiating miRs		
	T	PT	matched	paired t-test	2 samples t-test	CMSP	combined	#total	#up	#down
**$HER2^+$**	6	6	6	0.1	-	0.1	0.009	22	12	10
**Basal-like**	8	7	7	0.03	0.03	0.1	2×10^{-4}	31	18	13
**Luminal**	47	46	43	2×10^{-6}	2×10^{-6}	2×10^{-8}	1×10^{-20}	33	20	13

Supporting Information Table S2

For each subtype of breast cancer (HER2+/Basal-like/Luminal), we show the number of tumors (T), peritumors (PT) and matched samples pairs; the significance level used as threshold for q-value for FDR of each statistical test (paired t-test, 2-samples t-test, comparison of matched sample pairs (CMSP) and the combined p-value (see Methods)). The total number of miRs which passed these criteria, identified as differentiating tumor from peritumor samples, with specification of the number of miRs that were up or down regulated in the tumor tissue.

Supporting Information Table S3A Biagioni et al

A

microRNAs differentiating tumor and peritumor samples HER2+ tumors								
	Paired t-test		2 samples t-test		CMSP		Combined	
	p-value	q-value	p-value	q-value	p-value	q-value	p-value	q-value
hsa-miR-100	0.0053	0.080	-	-	0.0071	0.055	4.3E-04	0.0050
hsa-miR-106b	0.0012	0.060	-	-	0.0128	0.082	1.8E-04	0.0029
hsa-miR-10b*	0.0014	0.060	-	-	1.1E-04	0.007	2.5E-06	0.0002
hsa-miR-133b	0.0055	0.080	-	-	0.0190	0.100	0.0011	0.0085
hsa-miR-139-5p	0.0035	0.060	-	-	5.5E-05	0.005	3.2E-06	0.0002
hsa-miR-145	0.0025	0.060	-	-	0.0048	0.049	1.5E-04	0.0026
hsa-miR-18a	0.0027	0.060	-	-	2.1E-05	0.004	9.8E-07	0.0001
hsa-miR-18b	0.0021	0.060	-	-	6.6E-04	0.017	2.0E-05	0.0010
hsa-miR-198	0.0023	0.060	-	-	0.0201	0.100	5.1E-04	0.0055
hsa-miR-210	0.0085	0.100	-	-	0.0023	0.030	2.3E-04	0.0034
hsa-miR-301a	0.0032	0.060	-	-	1.2E-05	0.004	6.7E-07	0.0001
hsa-miR-32	0.0075	0.093	-	-	0.0019	0.029	1.7E-04	0.0029
hsa-miR-331-3p	6.7E-04	0.057	-	-	0.0049	0.049	4.5E-05	0.0016
hsa-miR-424*	0.0073	0.093	-	-	7.2E-04	0.017	6.9E-05	0.0017
hsa-miR-425	0.0097	0.100	-	-	0.0055	0.049	5.8E-04	0.0057
hsa-miR-429	8.2E-04	0.057	-	-	2.3E-04	0.008	3.2E-06	0.0002
hsa-miR-454	0.0034	0.060	-	-	0.0023	0.030	9.9E-05	0.0021
hsa-miR-486-5p	0.0032	0.060	-	-	0.0162	0.096	5.6E-04	0.0057
hsa-miR-542-5p	0.0023	0.060	-	-	0.0031	0.038	9.3E-05	0.0021
hsa-miR-7	6.8E-04	0.057	-	-	5.3E-05	0.005	6.5E-07	0.0001
hsa-miR-93	7.3E-04	0.057	-	-	0.0066	0.053	6.4E-05	0.0017
hsa-miR-936	0.0032	0.060	-	-	0.0054	0.049	2.1E-04	0.0031

Supporting Information Table S3

miRs differentiating tumor from peritumor tissues in the various subtypes of breast cancer. For each subtype: (A) $HER2^+$, (B) Basal-like and (C) Luminal, we listed the differentiating miRs with their p-values and corresponding q-values obtained by the following statistical analyses: paired t-test, 2-samples t-test, comparison of matched sample pairs (CMSP) and the combined p-value which was calculated from all tests together (see Methods).

Supporting Information Table S3B Biagioni et al

B

microRNAs differentiating tumor and peritumor samples Basal-like tumors								
	Paired t-test		2 samples t-test		CMSP		Combined	
	p-value	q-value	p-value	q-value	p-value	q-value	p-value	q-value
hsa-miR-106a	0.0032	0.017	0.0016	0.017	0.0204	0.091	1.5E-05	1.1E-04
hsa-miR-106b	0.0016	0.012	1.4E-04	0.007	0.0280	0.100	1.2E-06	1.5E-05
hsa-miR-10b*	0.0050	0.024	4.6E-04	0.010	3.5E-06	2.5E-04	2.9E-09	1.4E-07
hsa-miR-1225-5p	0.0033	0.018	0.0033	0.029	0.0141	0.074	2.1E-05	1.3E-04
hsa-miR-128	0.0028	0.017	0.0032	0.029	0.0106	0.063	1.4E-05	1.0E-04
hsa-miR-133b	9.1E-04	0.012	0.0015	0.017	0.0083	0.056	2.2E-06	2.3E-05
hsa-miR-135a*	0.0016	0.012	7.1E-04	0.012	5.8E-04	0.009	1.6E-07	2.7E-06
hsa-miR-139-5p	0.0018	0.013	3.8E-04	0.010	5.5E-05	0.001	1.2E-08	4.6E-07
hsa-miR-146a	0.0018	0.013	1.5E-04	0.007	0.0036	0.029	2.3E-07	3.7E-06
hsa-miR-155	0.0037	0.019	0.0015	0.017	0.0018	0.020	1.9E-06	2.1E-05
hsa-miR-17	4.9E-04	0.011	1.1E-04	0.007	0.0013	0.016	2.0E-08	5.4E-07
hsa-miR-17*	3.7E-04	0.011	4.5E-04	0.010	0.0034	0.029	1.4E-07	2.6E-06
hsa-miR-18a	0.0019	0.013	4.6E-05	0.005	3.3E-06	2.5E-04	1.3E-10	1.4E-08
hsa-miR-18b	0.0034	0.018	1.6E-04	0.007	7.7E-05	0.002	1.3E-08	4.6E-07
hsa-miR-193b*	0.0061	0.028	0.0016	0.017	0.0126	0.069	1.7E-05	1.1E-04
hsa-miR-301a	0.0022	0.014	0.0011	0.014	5.0E-05	0.001	3.3E-08	8.3E-07
hsa-miR-30c-1*	8.2E-04	0.012	7.5E-04	0.012	0.0123	0.069	1.5E-06	1.7E-05
hsa-miR-340	0.0014	0.012	3.4E-04	0.010	0.0292	0.100	2.5E-06	2.5E-05
hsa-miR-362-5p	0.0017	0.012	6.5E-04	0.012	0.0067	0.047	1.4E-06	1.7E-05
hsa-miR-374a	8.3E-04	0.012	0.0022	0.021	0.0259	0.100	7.4E-06	6.0E-05
hsa-miR-425	0.0015	0.012	0.0015	0.017	0.0104	0.063	4.0E-06	3.5E-05
hsa-miR-454	4.7E-04	0.011	8.9E-06	0.002	3.4E-05	0.001	6.7E-11	1.4E-08
hsa-miR-455-3p	2.9E-04	0.011	9.7E-04	0.013	1.2E-06	2.0E-04	1.5E-10	1.4E-08
hsa-miR-513a-5p	0.0011	0.012	0.0019	0.019	7.6E-05	0.002	4.4E-08	9.6E-07
hsa-miR-513b	8.9E-04	0.012	1.3E-04	0.007	4.6E-06	2.7E-04	2.4E-10	1.4E-08
hsa-miR-513c	4.5E-04	0.011	1.9E-04	0.008	5.4E-06	2.7E-04	2.0E-10	1.4E-08
hsa-miR-630	8.7E-04	0.012	6.2E-04	0.012	1.3E-04	0.002	2.0E-08	5.4E-07
hsa-miR-654-5p	0.0027	0.017	8.2E-04	0.012	0.0153	0.078	5.7E-06	4.8E-05
hsa-miR-660	0.0013	0.012	0.0016	0.017	0.0291	0.100	9.2E-06	7.1E-05
hsa-miR-887	0.0056	0.026	3.7E-04	0.010	7.3E-05	0.002	4.2E-08	9.6E-07
hsa-miR-93	2.7E-04	0.011	5.8E-07	2.0E-04	0.0025	0.024	1.7E-10	1.4E-08

Supporting Information Table S3C Biagioni et al

C

microRNAs differentiating tumor and peritumor samples Luminal tumors								
	Paired t-test		2 samples t-test		CMSP		Combined	
	p-value	q-value	p-value	q-value	p-value	q-value	p-value	q-value
hsa-miR-10b*	3.2E-12	5.6E-11	2.3E-07	1.7E-06	<1E-20	1.8E-19	<1E-20	<1E-20
hsa-miR-135a*	7.8E-12	1.2E-10	5.2E-09	5.9E-08	<1E-20	1.8E-19	<1E-20	<1E-20
hsa-miR-139-5p	7.9E-14	2.5E-12	3.2E-19	5.6E-17	<1E-20	1.8E-19	<1E-20	<1E-20
hsa-miR-141	5.6E-13	1.2E-11	8.7E-10	1.4E-08	<1E-20	1.8E-19	<1E-20	<1E-20
hsa-miR-142-3p	1.8E-11	2.5E-10	1.3E-08	1.2E-07	<1E-20	1.8E-19	<1E-20	<1E-20
hsa-miR-142-5p	6.9E-11	6.5E-10	9.8E-10	1.4E-08	1.9E-11	2.3E-10	<1E-20	<1E-20
hsa-miR-182	1.1E-15	1.2E-13	2.1E-17	1.8E-15	<1E-20	1.8E-19	<1E-20	<1E-20
hsa-miR-183	3.6E-17	6.2E-15	7.2E-18	8.3E-16	<1E-20	1.8E-19	<1E-20	<1E-20
hsa-miR-192	7.6E-11	7.0E-10	3.9E-09	4.7E-08	4.3E-09	3.5E-08	<1E-20	<1E-20
hsa-miR-193b*	2.6E-14	1.1E-12	5.0E-08	4.1E-07	3.2E-09	2.7E-08	<1E-20	<1E-20
hsa-miR-200a	4.3E-11	4.9E-10	1.1E-08	1.1E-07	<1E-20	1.8E-19	<1E-20	<1E-20
hsa-miR-200b	3.4E-11	4.0E-10	1.3E-08	1.2E-07	<1E-20	1.8E-19	<1E-20	<1E-20
hsa-miR-200c	6.8E-11	6.5E-10	2.0E-07	1.4E-06	1.3E-14	1.9E-13	<1E-20	<1E-20
hsa-miR-21*	2.0E-11	2.6E-10	2.0E-11	4.5E-10	8.8E-10	8.7E-09	<1E-20	<1E-20
hsa-miR-224	2.6E-11	3.3E-10	3.8E-13	1.2E-11	<1E-20	1.8E-19	<1E-20	<1E-20
hsa-miR-301a	2.8E-10	2.2E-09	3.7E-09	4.6E-08	3.9E-15	6.0E-14	<1E-20	<1E-20
hsa-miR-32	2.2E-12	4.0E-11	2.1E-11	4.5E-10	3.0E-15	5.0E-14	<1E-20	<1E-20
hsa-miR-33a	3.4E-10	2.5E-09	2.6E-09	3.6E-08	1.5E-09	1.4E-08	<1E-20	<1E-20
hsa-miR-342-5p	2.3E-14	1.1E-12	8.7E-17	6.1E-15	<1E-20	1.8E-19	<1E-20	<1E-20
hsa-miR-375	2.8E-15	2.5E-13	3.3E-11	6.4E-10	<1E-20	1.8E-19	<1E-20	<1E-20
hsa-miR-378	3.1E-18	1.1E-15	5.5E-20	1.9E-17	2.2E-16	3.9E-15	<1E-20	<1E-20
hsa-miR-425	2.5E-14	1.1E-12	1.1E-11	2.7E-10	2.1E-09	1.9E-08	<1E-20	<1E-20
hsa-miR-429	1.1E-14	7.5E-13	6.3E-12	1.7E-10	<1E-20	1.8E-19	<1E-20	<1E-20
hsa-miR-452	9.0E-10	6.0E-09	6.5E-12	1.7E-10	2.2E-10	2.3E-09	<1E-20	<1E-20
hsa-miR-454	2.7E-09	1.5E-08	1.7E-08	1.5E-07	4.0E-11	4.5E-10	<1E-20	<1E-20
hsa-miR-486-5p	3.5E-13	7.6E-12	2.7E-13	9.3E-12	1.2E-13	1.6E-12	<1E-20	<1E-20
hsa-miR-513b	1.2E-13	3.1E-12	1.2E-07	9.2E-07	<1E-20	1.8E-19	<1E-20	<1E-20
hsa-miR-513c	1.0E-13	3.0E-12	1.5E-07	1.1E-06	<1E-20	1.8E-19	<1E-20	<1E-20
hsa-miR-572	1.3E-09	8.3E-09	4.7E-09	5.4E-08	4.6E-09	3.7E-08	<1E-20	<1E-20
hsa-miR-630	6.1E-12	1.0E-10	6.1E-09	6.6E-08	1.5E-14	2.1E-13	<1E-20	<1E-20
hsa-miR-7	6.5E-12	1.0E-10	3.1E-11	6.3E-10	<1E-20	1.8E-19	<1E-20	<1E-20
hsa-miR-769-3p	3.0E-14	1.2E-12	4.4E-10	7.3E-09	3.0E-09	2.6E-08	<1E-20	<1E-20
hsa-miR-96	4.0E-14	1.4E-12	8.1E-14	3.5E-12	<1E-20	1.8E-19	<1E-20	<1E-20

Supporting Information Table S4 Biagioni et al

	N	%
**Number of patients**	59	
**Hystotype**		
Infiltrating Ductal Carcinoma	57	96,6
Infiltrating Lobular Carcinoma	2	3,4
**Grading**		
G1	3	5,1
G2	20	33,9
G3	35	59,3
unknown	1	1,7
**Tumor size**		
T1	26	44,1
T2	26	44,1
T3	7	11,8
**Nodal status**		
N0	36	61
N+	21	35,6
unknown	2	3,3
**Estrogen receptor**		
Positive (>10%)	3	5
Negative	56	95
**Progesteron receptor**		
Positive (>10%)	3	5
Negative	56	95
**HER2**		
Negative (score 0/1+)	29	49,2
Negative (score 2+ Not Amplified)	0	0
Positive (score 2+ Amplified + 3+)	30	50,8

Supporting Information Table S4

Clinical characteristics of breast cancer patients used for validation of microRNA expression profiles (FFPE tissues).

Supporting Information Table S5 Biagioni et al

A

KEGG pathway	# genes	p-value	FDR (%)
Cell cycle	7	3.8×10^{-8}	2.6×10^{-5}
Progesterone-mediated oocyte maturation	4	5.1×10^{-4}	0.35
p53 signaling pathway	3	0.007	4.92
Oocyte meiosis	3	0.019	11.99

B

Cell cycle target genes
CCNB3
PLK1
BUB1
CHEK1
CHEK2
CCNA2
CUL1

Supporting Information Table S5

(A) KEGG pathways enriched by the predicted target genes of miR-10b* (obtained from microcosm.org software) that were anti-correlated with the miR expression (in Enerly et al. dataset), and (B) the list of target genes enriched for cell cycle process.

Supporting Information Table S6 Biagioni et al

Primers used for 3' UTR cloning in psiCHEK2 plasmid

BUB1	Not I FW	5'-ATTTGCGGCCGCAATTTGGATATAGACAGTCC-3'
	Not I RV	5'-ATTTGCGGCCGCTTACATGGAAATATTCCATG-3'
PLK1	Not I FW	5'-CATCATGCGGCCGCTAGCTGCCCTCCCCTCCGGAC-3'
	Not I RV	5'-CATCATGCGGCCGCGAATATTCACATCTGTTTAATGTGCATAAAGCC-3'
CCNA2	Not I FW	5'-CATCATGCGGCCGCCAATGAAAGACTGCCTTTGTT-3'
	Not I RV	5'-CATCATGCGGCCGCAAGGTAACAAATTTCTGGTTTATTT-3'

Supporting Information Table S6

Primer sequences used to clone BUB1, PLK1 and CCNA2 3' UTR in psiCHEK2 plasmid.

Supporting Information Table S7 Biagioni et al

Primers used for 3ʼ UTR mutagenesis in psiCHEK2 plasmid

BUB1	mut FW	5ʼ - GCCGCAATTTGGATATAGACAGTCCTTAAAAATCACACTGTAAATATTCTAGATCTCAC TTTAAACCTGTTTT TTTTCATTTATTGTTTATGTAA - 3ʼ
	mut RV	5 ʻ- TTACATAAACAATAAATGAAAAAAAAACAGGTTTAAAGTGAGATCTAGAATATTTACA GTGTGATTTTTAAGGACTGTCTATATCCAAATTGCGG - 3ʼ
PLK1	PSICHEK 2 FW	5′ - CTTTTCCAACGCTATTGTCGAG - 3ʼ
	seed #1 Bam-HI RV	5′ - CGCGGATCCGGGAGGGGAGGGCAGC - 3ʼ
	seed #1 Bam-HI FW	5ʼ - CGCGGATCCGTGCCCTCCTCACTCCC - 3ʼ
	PSICHEK 2 RV	5′ - TCCGAAGACTCATTTAGATCCTC - 3ʼ
	seed #2 del 255-261 FW	5′ - AATTGTACAGAATATTTCTATTGAATTCTCCTTTCCTTGGCTTTATGC - 3ʼ
	seed #2 del 255-261 RV	5′ - GCATAAGCCAAGGAAAGGAGAATTCAATAGAAATATTCTGTACAATT - 3ʼ
CCNA2	del 176-183 FW	5ʼ -CTTTTTAAATGGTTTTAATTTGTATATCTTTTGTATATCTTAGATATTTGGCT AATTTTAAGTGGTTTTGTTAAA-3ʼ
	del 176-183 RV	5ʼ -TTTAACAAAACCACTTAAAATTAGCCAAATATCTAAGATATACAAAAGATA TACAAATTAAAACCATTTAAAAAG-3ʼ

Supporting Information Table S7

Primer sequences used for the mutagenesis of BUB1, PLK1 and CCNA2 wt 3ʼutr in psiCHEK2 plasmid.

Supporting Information Table S8 Biagioni et al

Primers and probes used for RT-qPCR in 5-Aza-dC experiment

GENE		SEQUENCE
pri-miR-10b	FW	5’ - CTGTAAGTACCTTCCCGCTTC -3’
	RV	5’ -CTTTCTTCTATCCGCCCACTC-3’
	probe	5’ - CCCCTTAGCCCACCACTTCCC - 3’
GAPDH	FW	5’ -GAGTCAACGGATTTGGTCGT-3’
	RV	5’ -GACAAGCTTCCCGTTCTCAG-3’

Supporting Information Table S8

Primer sequence for amplification of pri-miR-10b and GAPDH in 5’-Aza-dC experiment. All primer sets worked under identical quantitative PCR cycling conditions with similar efficiencies to obtain simultaneous amplification in the same run.

Supporting Information Table S9 Biagioni et al

Primers and probes used for RT-qPCR in MeDIP experiment

microRNA-10b* CpG island #1	**Forward** 5’ - CTGTAAGTACCTTCCCGCTTC - 3’<br>**Probe** 5’ - CCCCTTAGCCCACCACTTCCC - 3’<br>**Reverse** 5’ **-** CTTTCTTCTATCCGCCCACTC - 3’
microRNA-10b* CpG island #2	**Forward** 5’ - GGAATAGCCAGAGACCAAAGTG - 3’<br>**Probe** 5’ - CGACATGGAGACTAGAAGCAGCCG - 3’<br>**Reverse** 5’ - GGTAATAAAACAGAACGAGGCG - 3’
CpG free region (chr3:98760843+98761111)	**Forward** 5′ - TTGGCATTGATAATAACAGG - 3′<br>**Reverse** 5′ - AAGAAGTGAGTAGGGTAGGC - 3′

Supporting Information Table S9

Probes and primer sequences used for amplification of miR-10b*, miR-139-5p CpG islands and CpG free region in MeDIP experiment.

Supporting Information Table S10 Biagioni et al

Primers used for Bisulfite experiment

Forward primer 5’-3’	Reverse primer 5’-3’
ATTTTGGTAGAAGAATGAGGGAATT	TTCTTTTCAACACCCAAAAAATACT

Supporting Information Table S10

Primers used for the amplification of miR-10b* CpG island #2 in bisulfite experiment.

Supporting Information

Materials and Methods

Cell cultures and treatments

H1299, MCF7, MDA_MB_468, SKBR3 cells were cultured in DMEM medium (Invitrogen) supplemented with 10% fetal bovine serum, penicillin, and streptomycin. MCF10A cells were grown in Dulbecco's modified Eagle's medium–F12 (DMEM-F12) supplemented with antibiotics, insulin (10 mg/ml), hydrocortisone (0.5 mg/ml), heat inactivated horse serum [5% (vol/vol), Gibco BRL], and EGF (10 ng/ml). Cells were transfected with Lipofectamine 2000, following the manufacturer's instructions (Invitrogen) using 5 nM miRNA precursors and 5 nm miRNA precursor negative control #1 (Ambion). For siRNA experiments, MCF7 cells were transfected with siGFP duplex (AAGUUCAGCGUGUCCGGGGAG) (Benassi et al, 2012), siBUB1 #1 duplex (AAA UAC CAC AAU GAC CCA AGA) (Johnson et al, 2004), siBUB1 #2 duplex (AAA UAC CAC AAU GAC CCA AGA) (Meraldi & Sorger, 2005), siBUB1 #3 duplex (CCA UGG GAU UGG AAC CCU GU) (Tang et al, 2004), siPLK1 #1 duplex (CCA CCU UUU GAG ACU UCU U) (Judge et al, 2009), siPLK1 #2 duplex (AAG GGC GGC UUU GCC AAG UGC) (Xie et al, 2005), siPLK1 #3 duplex (GAU CAC CCU CCU UAA AUA U) (Spankuch et al, 2007), siCCNA2 #1 duplex (CCA UUG GUC CCU CUU GAU UU) (Wang et al, 2009), siCCNA2 #2 duplex (CAA GAA ACA AGU UCU GAG AAU) (designed with SciTool program on IDT website (eu.idtdna.com), siCCNA2 #3 duplex (AAC UAC AUU GAU AGG UUC CUG) (Mitra & Enders, 2004) at a final concentration of 40 nM. All siRNA were purchased from MWG Biotech company. AntagomiRs were designed as described (Krutzfeldt et al, 2005) and purchased from Fidelity System. Sequences were: "antagomiR-ctrl": 5'-U*A*CAGUACUUUUGUGUAGUACAAU*C*C*A*-Chol-3', "antagomiR-10b*": A*U*AUUCCCCUAGAAUCUGUG*A*C*U*-Chol-3'. All the bases are 2'-OMe modified, * represents a phosphorothioate linkage, and ''Chol'' represents linked cholesterol tail. MCF10A cells were transfected with 5nM antagomiR final concentration.

For 5-aza-2-deoxycytidine (5-Aza-dC; Sigma) cells were seeded at low density ($2x10^5$/60-mm dish) 16 h prior to treatment at the final concentrations of 20 μM. Medium containing fresh agent was changed every 24h for 96 h. Then 5-Aza-dC was removed and cells were culture for 24h hour more without treatment.

For the measurement of cell proliferation, cells transfected with miR-10b* oligo (described above) were incubated with BrdU (final concentration 20mM) at 72 h post transfection for 1 hour and harvested.

BrDU Immunofluorescence and Immunocytochemistry

Cells were seeded onto glass coverslips at $5x10^4$cells/ml in 6-well plates, transfected and fixed 72h later with Methanol/Acetone 1:1 in PBS for 15 minutes at -20°C. Cells were permeabilized with 0,1% Triton X-100 in PBS. After washing with PBS, the coverslips were incubated with BrDU antibody (DAKO) diluted in PBS for 1 hr at RT. Then they were three times with PBS/NaN_3, followed by incubation with Santa Cruz (mouse) conjugated secondary antibodies (Molecular Probes Inc) for 1 hour at RT. After washing 3X with PBS/NaN_3, the coverslips were counterstained with DAPI 3 min and mounted with Vectashield® (Vector Labs, CA).

For immunocytochemistry cells were incubated with peroxidase inhibitor before primary antibody incubation. The antibody used in this study was the anti-Ki67 antigen (diluted 1:100), supplied by DakoAS (Denmark), amplified by labeled streptavidinbiotin-peroxidase system obtained from Dako Cytomation. The color of immunostaining was developed by chromogenic substrate diaminobenzidine (100 mg%) and hydrogen peroxide (0.1%). After light counterstaining in Harry's hematoxylin, the slides were mounted and analyzed using light microscopy. Three independent experiments was considered for the quantification of positivity.

Tunel assay

The chromogenic in situ detection of apoptosis in formalin-fixed, paraffin-embedded mouse tumor tissues was performed by TdTmediated dUTP-biotin nick end labelling assay (TUNEL Apoptosis Detection Kit, GenScript USA Inc, Piscataway, USA). Following deparaffinization and rehydratation, 50 μl of TUNEL reaction mixture were applied to the sections, and the slides were incubated at 37°C for 60 min. The slides was then washed three times in PBS for 2 min, and the sections were incubated with Streptavidin-HRP Solution and then examined using a light microscope. Following TUNEL staining sections were scanned at 200X magnification to identify areas with the greatest apoptosis. Apoptosis of mouse tumor cells were counted in 8 high power fields (400X magnification) per section. Two independent observers performed the counts in blinded fashion. For each tumor counts were averaged to determine the number of apoptotic cells.

Soft agar assay

To assess the colony formation in soft agar, $1x10^4$ cells were inoculated into 0.5% agar containing DMEM supplemented with 10% FBS in a 6-well dish. After 2 weeks of incubation, the number of large colonies (over 0.125 mm in diameter) in each plate was scored. MCF7 line was tested in triplicate experiments.

Plasmid and transfection

The full length 3' UTR of the human BUB1, PLK1 and CCNA2 genes were amplified and inserted downstream of the Renilla luciferase gene into the NotI sites of the dual luciferase reporter plasmid psiCHECK-2 (Promega) (Supplementary Information Table S6). All constructs were verified by sequencing. BUB1, PLK1 and CCNA2 mutants were made with the QuikChange® site-directed mutagenesis kit (Stratagene) (Supplementary Information Table S7). PLK1 mutant was obtained performing a PCR inserting a BamHI site in correspondence of seed #1 site and then mutagenizing seed #2 with primers in Supplementary Information Table S7. Each of the mutants was sequenced to confirm the mutated products. H1299 cells were transfected using Lipofectamine 2000 (Invitrogen) with 100 ng of psiCHECK-2 reporter vector containing the different 3' UTR of putative target genes together with the human miR-10b* precursor or a control precursor (Ambion) at final concentrations of 5 nM. Firefly and Renilla luciferase activities were measured 36h post transfection using the Dual Luciferase Reporter Assay System (Promega) in the GloMax 96 Microplate Luminometer (Promega). Firefly luciferase was used to normalize the Renilla luciferase.

Lysate preparation and immunoblotting analyses

Cells were lysed in hypotonic lysis buffer (10mM Tris pH 8; 120mM NaCl; 15% glycerol; 0,5% Nonidet P-40; fresh DTT 2.5mM and fresh protease inhibitors). Extracts were sonicated for 20s and centrifuged at 14000 ×rpm for 15min to remove cell debris. Protein concentrations were determined by colorimetric assay (Bio-Rad). Western blotting was performed using the following primary antibodies: mouse monoclonal anti-GAPDH (Santa Cruz), mouse monoclonal anti-BUB1 (Millipore), rabbit polyclonal anti-BUB1 (Abcam), mouse monoclonal anti-PLK1 (Abcam), rabbit polyclonal anti-CCNA2 (Abgent) and rabbit polyclonal anti-CCNA2 (Santa Cruz). Secondary antibodies used were goat anti-mouse, goat anti-rabbit conjugated to horseradish peroxidase (Amersham). Immunostained bands were detected by chemiluminescent method (Pierce).

RNA extraction and reverse transcriptase reaction

Cells were harvested in TRIzol reagent (Invitrogen) and total RNA was isolated as the manufacturer's instructions. Five micrograms of total RNA were reverse-transcribed at 37 °C for 60 minutes in the presence of random hexamers and Moloney murine leukemia virus reverse transcriptase (Invitrogen). Polymerase Chain Reaction (PCR) analyses were carried out by using oligonucleotides specific for the genes listed in Supporting Information Table S8. pri-miR-10b and GAPDH expression were measured by real-time PCR using Sybr Green or TaqMan assays

respectively on a StepOne instrument (Applied Biosystems).

Identifying differentially expressed miRs between tumor and peritumor samples in the various subtypes of breast cancer Three statistical tests were used to define the group of differentially expressed miRs in each subtype: i) paired t-test ii) 2-samples t-test and iii) CMSP (see Methods). Since for most patients both the tumor and matched peritumor samples were available, it made sense to evaluate p-values using the paired t-test and the CMSP. The 2-samples t-test was also tried, in order to include also patients for whom only one of the samples was available. For patients with matched samples we decided to combine results of the paired t-test and CMSP, since their combination produced a more accurate signature than using each of them alone. The reason is that the two tests complement each other. The paired t-test statistic is based on the mean of the differences between paired observations across all patients, while in CMSP a p-value is assigned for each patient first, and then the p-values from all patients are combined. Moreover in CMSP noise is estimated on the basis of 16 replicate samples, whereas in the paired t-test it is calculated by the differences' standard deviation. Hence, CMSP has more statistical power, and therefore it captures better consistent differences across the majority of the patients, even when the differences are low. On the other hand, the paired t-test filters out miRs which are extremely differently expressed, but only in a small subset of the patients, while in the rest of the patients there is no change in their expression level (a situation that occurs frequently due to "contamination" of the peritumor tissue with cells of other than epithelial origin, in which case the differences between the paired samples result from variation between cells of different origin, and not from variation between the tumor and epithelial peritumor tissue).

Sorting Points Into Neighborhood (SPIN) SPIN is an unsupervised method for sorting and visualization of multidimensional data (Tsafrir et al, 2005). The algorithm creates iteratively an optimal ordering of the objects being studied, such that the distances are small along the diagonal of the pairwise distance matrix and increase as they move away from it. This ordering allows for finding groups of miRs that display similar expression patterns over a range of samples.

cDNA synthesis and RT q-PCR

RT q-PCR quantification of miRNA expression was performed using TaqMan MicroRNA® Assays (Applied Biosystems, Foster City, CA, USA) according to the manufacturer's protocol. Small amount of RNA (10 ng) was reverse-transcribed using the TaqMan microRNA Reverse

Transcription Kit (Applied Biosystem). PCR reactions were carried out in final volumes of 7,5 µl using an ABI Prism 7000 Sequence Detection System (Applied Biosystems). Reactions were initiated with a 10 minute incubation at 95°C followed by 40 cycles of 95°C for 15 seconds and 60°C for 60 seconds. RTq-PCR quantification of miRNA expression was performed using TaqMan MicroRNA® Assays (Applied Biosystems) according to the manufacturer's protocol RNU49 was used as endogenous controls to standardize miRNA expression. All reactions were performed in duplicate.

Immunohistochemistry

Ten breast cancer and the correspondent peritumoral tissues were randomly selected from the 61 cases already evaluated for miRNA profiles. Formalin-fixed paraffin-embedded BC specimens were cut on SuperFrost Plus slides (Menzel-Glaser, Braunschweig, Germany). Two micron-thick paraffin-embedded sections were stained with a streptavidin-enhanced immunoperoxidase technique (Supersensitive Multilink, Novocastra, Menarini Florence, Italy) in an automated autostainer (Bond Max, Menarini) using the following reagents: anti-BUB1 monoclonal antibody (moab) (clone 14H5, 1:100; Millipore, Temecula, CA, USA), anti-PLK1 moab (clone 36-298, 1:200, Abcam, Cambridge, UK), CCNA2 rabbit polyclonal antibody (1:50; ABGENT, San Diego, CA, USA). Moreover in five representative injected miR-10b* and control mimic mice ki67 and Cyclin D1 expression were tested using the following antibodies: anti-Ki67 antigen (diluted 1:100), supplied by DakoAS (Denmark) and anti-Human Cyclin D1, monoclonal Rabbit antibody, clone SP4 supplied by Dako AS. The pH 6 citrate buffer antigen retrieval protocol was applied for the anti-BUB1 while for the others antibodies was applied the pH 8 buffer antigen retrieval protocol. Diaminobenzidine (Menarini) was used as chromogenic substrate.

The immunostaining was considered positive when at least 10% of the neoplastic cells showed a distinct cytoplasmic (BUB1 and CCNA2) and/or nuclear/cytoplasmic (PLK1) immunoreactivity. Evaluation of the immunohistochemical results, blinded to all patient data, was performed independently by two investigators (MM, ADB).

References Supporting Information

Benassi B, Flavin R, Marchionni L, Zanata S, Pan Y, Chowdhury D, Marani M, Strano S, Muti P, Blandino G et al (2012) MYC is activated by USP2a-mediated modulation of microRNAs in prostate cancer. Cancer discovery 2**:** 236-247

Johnson VL, Scott MI, Holt SV, Hussein D, Taylor SS (2004) Bub1 is required for kinetochore localization of BubR1, Cenp-E, Cenp-F and Mad2, and chromosome congression. J Cell Sci 117**:** 1577-1589

Judge AD, Robbins M, Tavakoli I, Levi J, Hu L, Fronda A, Ambegia E, McClintock K, MacLachlan I (2009) Confirming the RNAi-mediated mechanism of action of siRNA-based cancer therapeutics in mice. J Clin Invest 119**:** 661-673

Krutzfeldt J, Rajewsky N, Braich R, Rajeev KG, Tuschl T, Manoharan M, Stoffel M (2005) Silencing of microRNAs in vivo with 'antagomirs'. Nature 438**:** 685-689

Meraldi P, Sorger PK (2005) A dual role for Bub1 in the spindle checkpoint and chromosome congression. EMBO J 24**:** 1621-1633

Mitra J, Enders GH (2004) Cyclin A/Cdk2 complexes regulate activation of Cdk1 and Cdc25 phosphatases in human cells. Oncogene 23**:** 3361-3367

Spankuch B, Kurunci-Csacsko E, Kaufmann M, Strebhardt K (2007) Rational combinations of siRNAs targeting Plk1 with breast cancer drugs. Oncogene 26**:** 5793-5807

Tang Z, Sun Y, Harley SE, Zou H, Yu H (2004) Human Bub1 protects centromeric sister-chromatid cohesion through Shugoshin during mitosis. Proc Natl Acad Sci U S A 101**:** 18012-18017

Tsafrir D, Tsafrir I, Ein-Dor L, Zuk O, Notterman DA, Domany E (2005) Sorting points into neighborhoods (SPIN): data analysis and visualization by ordering distance matrices. Bioinformatics 21**:** 2301-2308

Wang X, Song Y, Ren J, Qu X (2009) Knocking-down cyclin A(2) by siRNA suppresses apoptosis and switches differentiation pathways in K562 cells upon administration with doxorubicin. PLoS One 4**:** e6665

Xie S, Xie B, Lee MY, Dai W (2005) Regulation of cell cycle checkpoints by polo-like kinases. Oncogene 24**:** 277-286
